# Caspofungin-induced β(1,3)-glucan exposure in *Candida albicans* is driven by increased chitin levels

**DOI:** 10.1128/mbio.00074-23

**Published:** 2023-06-28

**Authors:** Andrew S. Wagner, Stephen W. Lumsdaine, Mikayla M. Mangrum, Todd B. Reynolds

**Affiliations:** 1 Department of Microbiology, University of Tennessee, Knoxville, Tennessee, USA; Duke University, Durham, North Carolina, USA

**Keywords:** *Candida albicans*, caspofungin, chitin, β-glucan, unmasking

## Abstract

To successfully induce disease, *Candida albicans* must effectively evade the host immune system. One mechanism used by *C. albicans* to achieve this is to mask immunogenic β(1,3)-glucan epitopes within its cell wall under an outer layer of mannosylated glycoproteins. Consequently, induction of β(1,3)-glucan exposure (unmasking) *via* genetic or chemical manipulation increases fungal recognition by host immune cells *in vitro* and attenuates disease during systemic infection in mice. Treatment with the echinocandin caspofungin is one of the most potent drivers of β(1,3)-glucan exposure. Several reports using murine infection models suggest a role for the immune system, and specifically host β(1,3)-glucan receptors, in mediating the efficacy of echinocandin treatment *in vivo*. However, the mechanism by which caspofungin-induced unmasking occurs is not well understood. In this report, we show that foci of unmasking co-localize with areas of increased chitin within the yeast cell wall in response to caspofungin, and that inhibition of chitin synthesis *via* nikkomycin Z attenuates caspofungin-induced β(1,3)-glucan exposure. Furthermore, we find that both the calcineurin and Mkc1 mitogen-activated protein kinase pathways work synergistically to regulate β(1,3)-glucan exposure and chitin synthesis in response to drug treatment. When either of these pathways are interrupted, it results in a bimodal population of cells containing either high or low chitin content. Importantly, increased unmasking correlates with increased chitin content within these cells. Microscopy further indicates that caspofungin-induced unmasking correlates with actively growing cells. Collectively, our work presents a model in which chitin synthesis induces unmasking within the cell wall in response to caspofungin in growing cells.

**IMPORTANCE:**

Systemic candidiasis has reported mortality rates ranging from 20% to 40%. The echinocandins, including caspofungin, are first-line antifungals used to treat systemic candidiasis. However, studies in mice have shown that echinocandin efficacy relies on both its cidal impacts on *Candida albicans*, as well as a functional immune system to successfully clear invading fungi. In addition to direct *C. albicans* killing, caspofungin increases exposure (unmasking) of immunogenic β(1,3)-glucan moieties. To evade immune detection, β(1,3)-glucan is normally masked within the *C. albicans* cell wall. Consequently, unmasked β(1,3)-glucan renders these cells more visible to the host immune system and attenuates disease progression. Therefore, discovery of how caspofungin-induced unmasking occurs is needed to elucidate how the drug facilitates host immune system-mediated clearance *in vivo*. We report a strong and consistent correlation between chitin deposition and unmasking in response to caspofungin and propose a model in which altered chitin synthesis drives increased unmasking during drug exposure.

## INTRODUCTION

The fungal cell wall is a dynamic structure that sits at the interface between pathogen and host. In *Candida albicans*, the cell wall consists of three major layers: a basal chitin layer, followed by a central layer of β(1,3) and β(1,6)-glucan polymers, and an outer layer of mannosylated glycoproteins (1, 2). These cell wall epitopes are detected by multiple pattern recognition receptors (PRRs) that are expressed within various host cell types to facilitate appropriate responses to invading fungal pathogens. For example, mannoproteins in the outer cell wall can be recognized by at least six different host PRRs, including the C-type lectin receptor dectin-2 (3, 4), galectin-3 (5 - 7), mannose receptor (8, 9), dendritic cell-specific intracellular adhesion molecule 3-grabbing non-integrin (9), and the toll-like receptors TLR2 (10) and TLR4 (8). Exposed (1,3)-glucan is recognized by different receptors expressed on both hematopoietic and non-hematopoietic cell lines, including dectin-1 (11 - 16), complement receptor 3 (17, 18), and the ephrin type-A receptor 2 (19, 20). Chitin recognition is not as well understood, but evidence suggests that the intracellular receptors TLR2 and NOD2 can recognize chitin, as can the extracellular epithelial cell receptor LYSMD3 (21, 22).

Among the cell wall components, β(1,3)-glucan is highly pro-inflammatory, and its exposure has been found to occur during disease progression. However, increased β(1,3)-glucan exposure can attenuate virulence. Given these observations, inappropriately exposing β(1,3)-glucan moieties (in a process termed unmasking) has begun to gain attention as a potential immunotherapeutic strategy (23 - 25). Accordingly, multiple exogenous stimuli and genetic mutations have been identified that impact the unmasking state of the cell in ways that favor the pathogen or the host. For example, exposure to exogenous lactate, calcium, hypoxia, or iron limitation reduces β(1,3)-glucan exposure (masking) (24, 26 - 29) and is suggested to aid the pathogen. In contrast, growth in acidic pH or in the presence of the short-chain fatty acid acetate or butyrate induces unmasking (28, 30, 31). Additionally, deletion of the phosphatidylserine synthase *CHO1* (32, 33), the glycosyltransferase *KRE5* (34), the endoglucanase *ENG1* (35), or the outer cell wall protein *FGR41* (29) induces unmasking and attenuates systemic disease progression. Similarly, hyperactivation of the Cek1 mitogen-activated protein kinase (MAPK) pathway also increases β-glucan exposure and attenuates virulence in a host immune system-dependent manner (36 - 39). Yet, among all stimuli, exposure to the echinocandin caspofungin has been found to be one of the most potent drivers of unmasking (34, 40, 41). However, little is known about how β-glucan exposure occurs during caspofungin treatment, or the impact that this may have on drug efficacy.

Echinocandins function by inhibiting the β(1,3)-glucan synthase enzymes Fks1 and Fks2, and are largely thought to function in a fungicidal manner against *C. albicans* (42). However, evidence in mice suggests that the full mechanism of action for these drugs during treatment is also dependent on a functional host immune response. For example, when comparing anidulafungin efficacy in immunocompetent and neutropenic mice systemically infected with *C. albicans,* the survival rate was found to be much lower in the immunosuppressed mice, regardless of the dosage of anidulafungin (43). Furthermore, the ability of caspofungin to reduce kidney fungal burden following systemic *C. albicans* infection in immunocompetent and dectin-1^−/−^ mice also showed a reliance on this β(1,3)-glucan receptor for effective drug-mediated clearance (44). These host-dependent mechanisms of action during *in vivo* drug treatment are presumably the consequences of drug-induced unmasking. Therefore, understanding how caspofungin induces unmasking is not only clinically relevant, but also allows for a better understanding of how β-glucan exposure is regulated by *C. albicans* in response to exogenous stimuli.

Treatment with caspofungin activates at least four signal transduction pathways: the Cek1, Mkc1, and Hog1 MAPK pathways, as well the calcineurin pathway (45 - 48). In addition to unmasking, increased chitin synthesis is also observed, and chitin serves as a compensatory mechanism for inhibition of the β(1,3)-glucan synthases to provide structural support for the cell wall (48 - 50). Higher basal chitin content reduces caspofungin sensitivity (51 - 53). Presumably, both chitin synthesis and unmasking are mediated by one or more of these signaling pathways. However, delineation of signal transduction pathways that mediate unmasking specifically and the relationship that increased β-glucan exposure has with chitin content during caspofungin treatment remains to be fully elucidated. We have previously shown that calcineurin, but not the Cek1 MAPK pathway, is needed to induce the full levels of unmasking observed during caspofungin treatment (29). In this report, we show that foci of exposed β(1,3)-glucan predominantly co-localize with areas of increased chitin deposition during capsofungin exposure, and that simultaneous treatment with the chitin synthase inhibitor nikkomycin Z attenuates both caspofungin-induced chitin synthesis and unmasking. We also report that both the calcineurin and Mkc1 signal transduction pathways play an important role in regulating unmasking and chitin synthesis in response to drug treatment. Furthermore, we show that a bimodal population of high and low chitin-containing cells is observed during caspofungin treatment in calcineurin-inhibited and *mkc1Δ/Δ* cells, and that the chitin content in the cell wall directly correlates with the levels of β-glucan exposure following drug treatment. Accordingly, we propose a model in which chitin serves as a driver to induce unmasking in response to caspofungin.

## RESULTS

### Foci of increased chitin deposition co-localize with unmasked β(1,3)-glucan during caspofungin exposure

**Fig 1:**
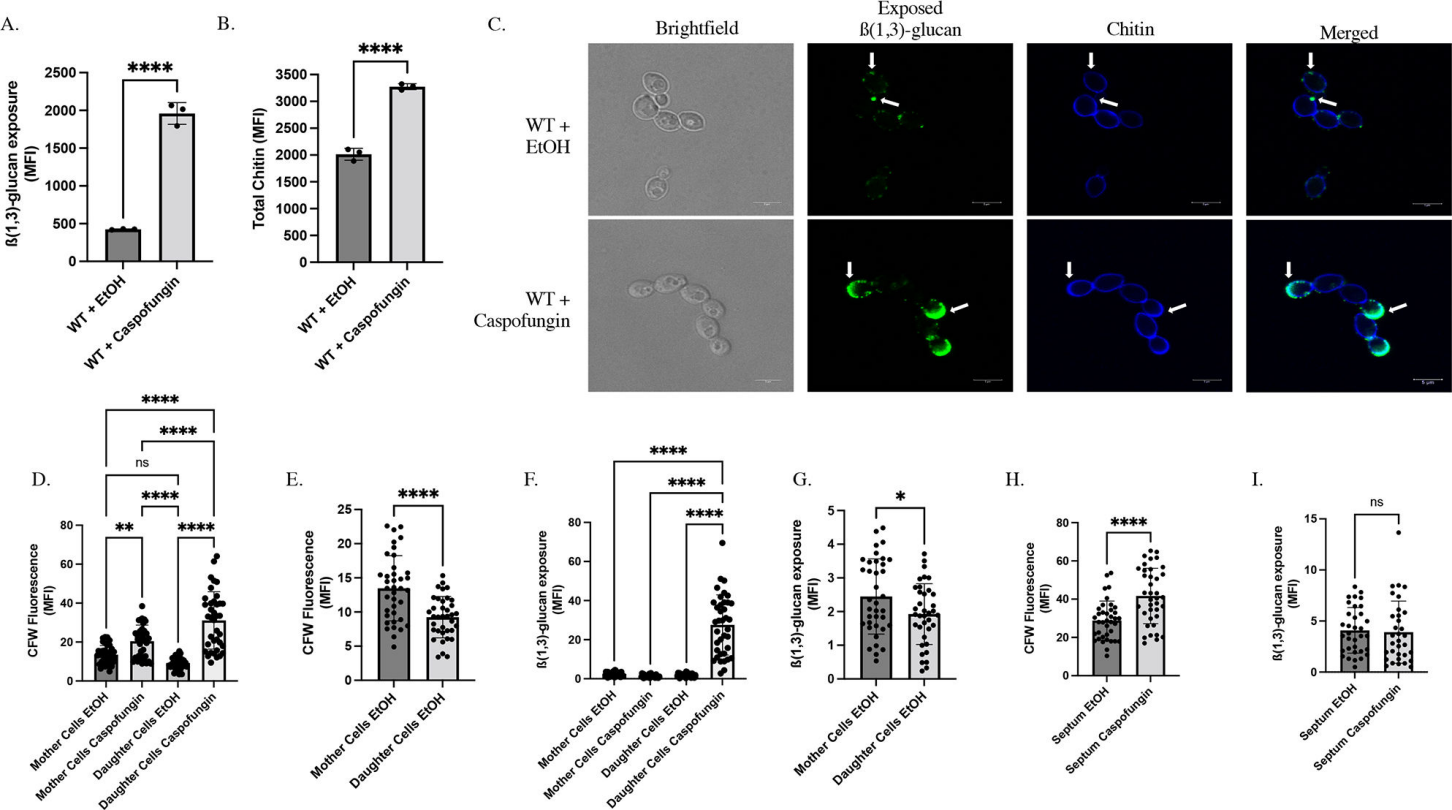
Areas of increased chitin co-localize with unmasked foci in growing yeast cells in response to caspofungin. (**A–B**) SC5314-derived *LEU2/leu2∆* WT cells (AWY006) grown to mid-log phase were exposed to 46.9 ng/mL of caspofungin for 30 minutes and then stained with anti-β(1,3)-glucan antibody and a phycoerythrin-conjugated secondary antibody, along with calcofluor white (CFW) to assess the levels of β(1,3)-glucan exposure and total chitin by flow cytometry, respectively. (**A**) β(1,3)-glucan unmasking in WT cells treated with sublethal concentrations of caspofungin. (**B**) CFW staining of WT cells treated with sublethal concentrations of caspofungin (****P < 0.0001 by Student’s t-test*; n* = 3 biological replicates). (**C**) Representative confocal microscopy of mid-log phase cells treated with an ethanol solvent control or caspofungin. White arrows highlight examples of unmasking across images (scale bar indicates 5 µm). (**D–I**) CFW staining and β(1,3)-glucan unmasking quantifications of microscopy images of mother cells, daughter cells, and septa of budding yeast (*n* = 38 budding cells analyzed per treatment). (**D**) CFW staining of WT cells treated with sublethal concentrations of caspofungin or an ethanol solvent control [**P < 0.005, ****P < 0.0001 by one-way analysis of variance (ANOVA)]. (**E**) CFW staining of WT cells treated with ethanol only. These data are the same data as were presented in Fig. 1D, but were highlighted here for specific analysis of total chitin levels in basal growth conditions (****P < 0.0001 by Student’s t-test). (**F**) Unmasking of WT cells treated with sublethal concentrations of caspofungin or an ethanol solvent control (****P < 0.0001 by Kruskal–Wallis test). (**G**) Unmasking of WT cells treated with ethanol only. These data are the same data as were presented in Fig. 1F, but were highlighted here for specific analysis of β(1,3)-glucan exposure levels in basal growth conditions (*P < 0.05 by Student’s t-test). (**H**) CFW staining of the septa of cells treated with caspofungin or an ethanol solvent control (****P < 0.0001 by Student’s t-test). (**I**) Unmasking of the septa of cells treated with caspofungin or an ethanol solvent control. MFI, median fluorescent intensity; ns, not significant by Mann–Whitney test.

The mechanism by which caspofungin induces unmasking is currently unknown. However, yeast cells exposed to caspofungin exhibit both unmasking (34, 40, 41) and increases in total chitin (48 - 50). To further explore how these correlative phenomena relate to each other, flow cytometry was performed on SC5314-derived *LEU2/leu2∆* wild-type (WT) cells (Table S1) to examine both simultaneously. This experiment revealed that caspofungin treatment for 30 minutes at a sublethal concentration [46.9 ng/mL; corresponding to 3/8 of the minimum inhibitory concentration (MIC) of our strain] of drug induces both an increase in total chitin levels and β(1,3)-glucan unmasking (Fig. 1A and B; Fig. S1). We hypothesized that foci of unmasked β(1,3)-glucan would co-localize with areas of increased chitin deposition within the cell wall. Microscopy revealed a scattered distribution of unmasked β(1,3)-glucan foci throughout the cell wall of yeast treated with an ethanol solvent control that had minimal overlap with areas of dense chitin content (Fig. 1C). However, yeast cells that were exposed to caspofungin predominantly showed a robust increase in unmasking at the tip of what appeared to be the smaller budding daughter cells. Correspondingly, this co-localized with areas of increased chitin deposition at these poles.

To quantify the relationship more thoroughly between unmasking and chitin content in budding cells, we measured the mean fluorescent intensity for both β-glucan and calcofluor white (CFW) staining in the larger mother cells, the smaller daughter cells, and at the septa (Fig. 1D through I). In the solvent control samples, total chitin levels and unmasking were higher in the mother cells than budding daughter cells (Fig. 1E and G), which supports the observation that mother cells display more exposed β(1,3)-glucan during active growth (54). However, upon caspofungin treatment, these trends were reversed, and the smaller daughter cells displayed significantly higher levels of both chitin and unmasking when compared to mother cells within the caspofungin-treated samples or the solvent controls (Fig. 1D and F). Furthermore, although the septa showed the highest levels of both basal chitin and β-glucan exposure in the solvent controls, the levels of unmasking at the septa were not significantly altered during caspofungin treatment (Fig. 1H and I). Thus, it appears that areas of increased unmasking co-localize with increased chitin deposition within the cell wall of budding daughter cells, and this may represent a mechanism by which the cell wall is perturbed to increase β-glucan exposure.

*C. albicans* can grow as hyphae, which also exhibit unmasking in response to caspofungin (40). To determine how hyphal cells were impacted for unmasking during active growth, they were exposed to caspofungin. Interestingly, caspofungin-induced unmasking in hyphal cells also appeared to occur primarily on the cell wall between the apical hyphal tip and the most distal septum from the basal hyphal head (Fig. S2). This part of the hyphal cell also appears to specifically increase in chitin following caspofungin exposure, but its deposition appears to be more uniform across the lateral cell wall, rather than specifically co-localizing with pockets of increased β-glucan exposure.

### Nikkomycin Z treatment attenuates caspofungin-induced chitin synthesis and unmasking in yeast cells

**Fig 2:**
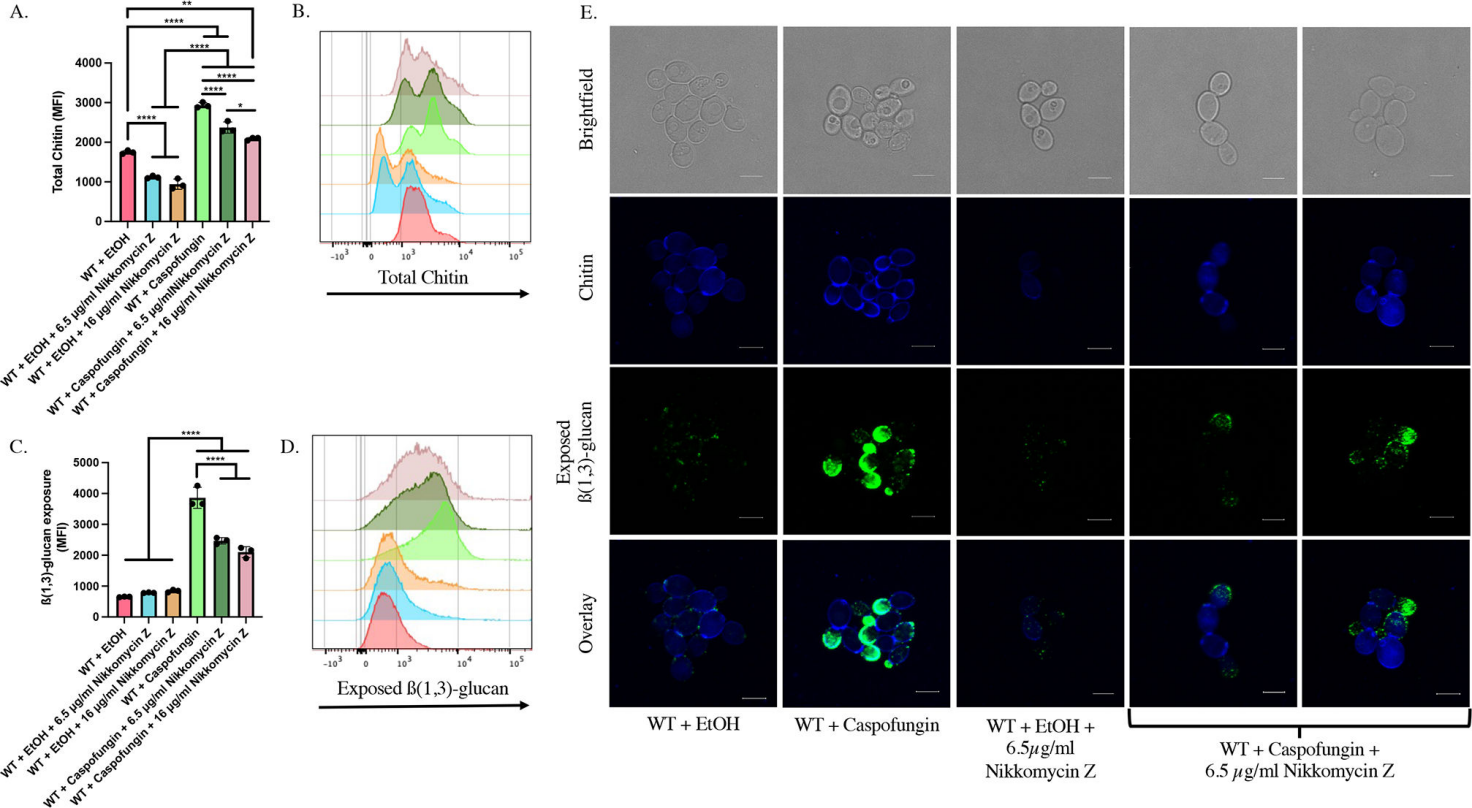
Nikkomycin Z treatment attenuates caspofungin-induced unmasking in yeast cells. SC5314-derived *LEU2/leu2∆* (AWY006) WT cells grown to mid-log phase were exposed to 46.9 ng/mL of caspofungin and varying concentrations of nikkomycin Z (6.5 µg/mL or 16.5 µg/mL), or appropriate solvent controls, for 30 minutes. β(1,3)-glucan exposure and total chitin levels were then assessed by flow cytometry. (**A**) CFW staining of nikkomycin Z- and caspofungin-treated samples (*P < 0.05, **P < 0.005, ****P < 0.0001, by one-way ANOVA*; n* = 3 biological replicates). (**B**) Representative histogram of impact that nikkomycin Z treatment has on caspofungin-induced chitin synthesis. Colors of each sample match those shown in Fig. 2A. (**C**) β(1,3)-glucan exposure of nikkomycin Z- and caspofungin-treated samples (****P < 0.0001 by one-way ANOVA*; n* = 3 biological replicates). (**D**) Representative histogram of impact that nikkomycin Z treatment has on caspofungin-induced unmasking. Colors of each sample match those shown in Fig. 2C. (**E**) Representative microscopy images of nikkomycin Z- and caspofungin-treated cells (scale bar indicates 5 µm).

Given the observation that increased chitin deposition co-localizes with foci of unmasking during caspofungin treatment, we hypothesized that inhibition of chitin synthesis would attenuate caspofungin-induced β(1,3)-glucan exposure. To test this, we treated cells with varying concentrations of the chitin synthase inhibitor nikkomycin Z simultaneously with caspofungin addition. Nikkomycin Z treatment caused a dose-dependent reduction in total chitin during caspofungin addition (Fig. 2A and B). Furthermore, this corresponded to a significant reduction in β(1,3)-glucan exposure (Fig. 2C and D). Microscopy confirmed that many cells in the nikkomycin Z- plus caspofungin-treated samples exhibited reduced chitin and unmasking within budding daughter cells (Fig. 2E). However, it is important to note that not all cells showed a robust reduction in unmasking and that a range of β-glucan exposures was observed in the group treated with both drugs. Given the wide distribution observed in the nikkomycin Z- and caspofungin-treated population during flow cytometry (Fig. 2D), this is not entirely surprising. Nevertheless, these data strongly support the model that altered chitin synthesis impacts β-glucan exposure in response to caspofungin.

### Calcineurin mediates chitin synthesis and unmasking in yeast cells in response to caspofungin treatment

**Fig 3:**
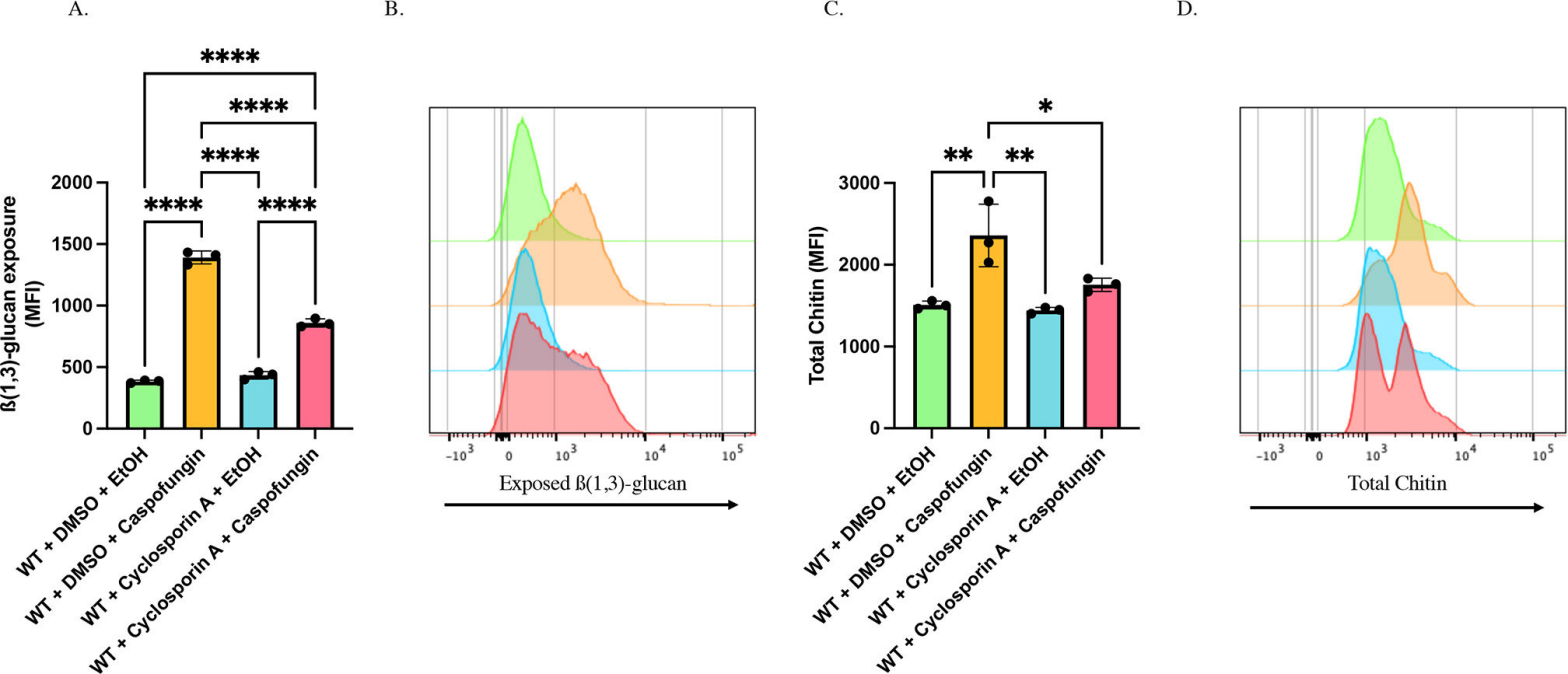
Calcineurin inhibition attenuates both unmasking and chitin synthesis in yeast cells in response to caspofungin exposure. SC5314-derived *LEU2/leu2∆* (AWY006) WT cells grown to mid-log phase in the presence of 100 µg/mL of cyclosporine A, or an appropriate volume of the DMSO solvent control, were exposed to 46.9 ng/mL of caspofungin (or EtOH solvent control) for 30 minutes. β(1,3)-glucan exposure and total chitin levels were then assessed by flow cytometry. (**A**) β(1,3)-glucan exposure of cyclosporine A- and caspofungin-treated cells (****P < 0.0001 by one-way ANOVA*; n* = 3 biological replicates). (**B**) Representative histogram of the impact that calcineurin inhibition has on caspofungin-induced unmasking. Colors of each sample match those shown in Fig. 2A. (**C**) CFW staining of cyclosporine A- and caspofungin-treated cells (*P < 0.01, **P < 0.005*; n* = 3 biological replicates). (**D**) Representative histogram of the impact that calcinuerin inhibition has on caspofungin-induced chitin synthesis. Colors of each sample match those shown in Fig. 2A and C.

We demonstrated that calcineurin is responsible, in part, for the unmasking observed during caspofungin treatment (29). Calcineurin has been implicated as a major regulator of chitin synthesis in response to caspofungin addition (48). Given the observation that inhibition of chitin synthesis can attenuate caspofungin-induced unmasking (Fig. 2), we hypothesized that calcineurin induces unmasking during caspofungin exposure by altering the chitin content within the yeast cell wall. In accordance with previous observations (29), calcineurin inhibition *via* cyclosporine A prior to caspofungin exposure significantly reduced unmasking (Fig. 3A and B). Total chitin content was also reduced during calcineurin inhibition (Fig. 3C) and followed the same trend as unmasking (Fig. 3A). Microscopy revealed a range of phenotypes where some cells showed moderately reduced levels of unmasking during cyclosporine A and caspofungin treatment, while other cells in the population showed comparable levels of unmasking to the caspofungin only control (Fig. S3). Viability plating revealed no differences in growth following drug treatment (Fig. S4), therefore suggesting that differences were not explained by cell death.

**Fig 4:**
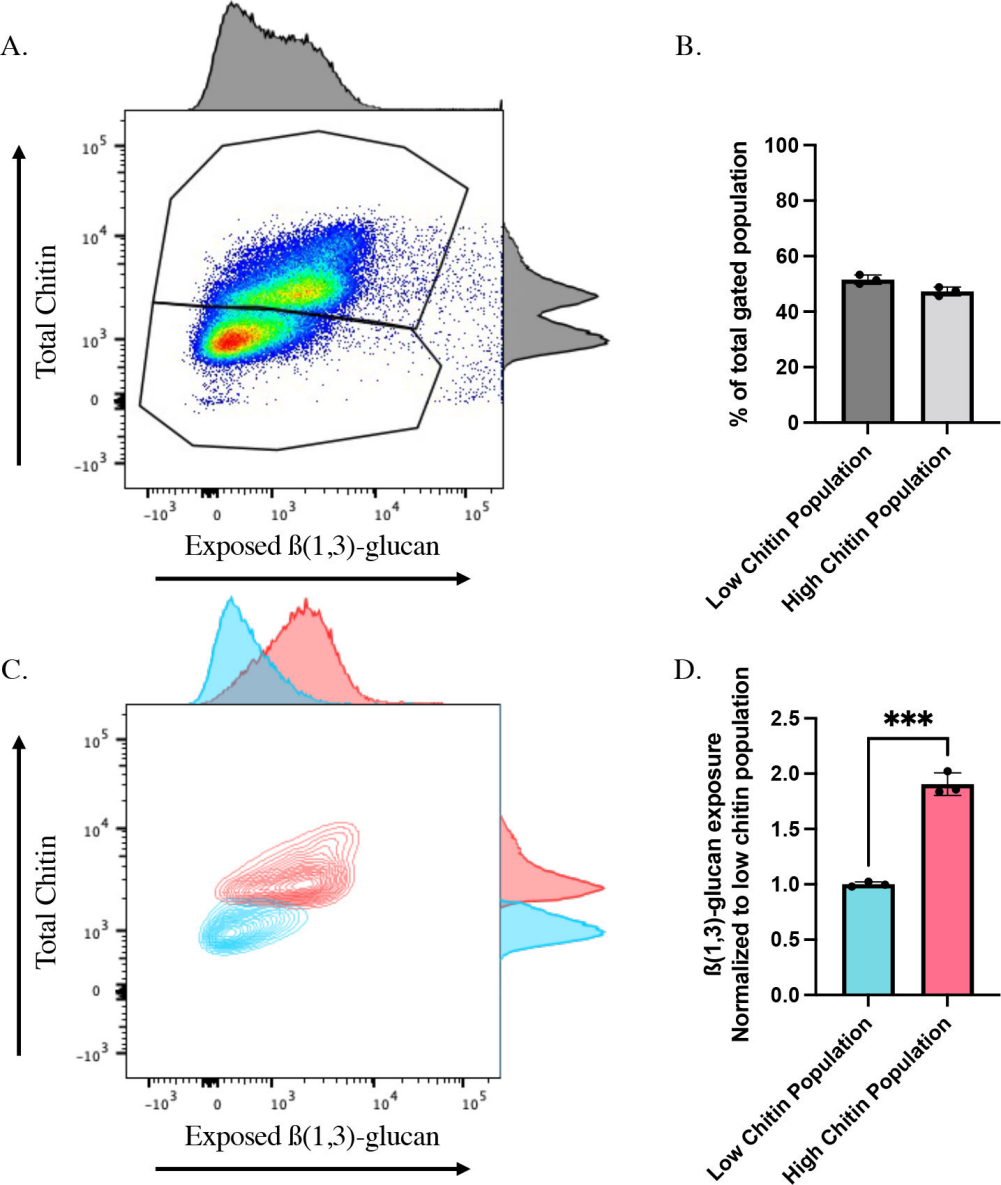
Chitin levels correlate with β(1,3)-glucan exposure following caspofungin treatment in calcineurin-inhibited yeast cells. SC5314-derived *LEU2/leu2∆* (AWY006) WT cells grown to mid-log phase in the presence of 100 µg/mL of cyclosporine A, or an appropriate volume of the DMSO solvent control, were exposed to 46.9 ng/mL of caspofungin (or EtOH solvent control) for 30 minutes, and β(1,3)-glucan exposure and total chitin levels were assessed by flow cytometry. (**A**) Representative scatter plot and adjunct histograms of cyclosporine A- and caspofungin-treated cells when assessing unmasking and total chitin. Gates represent populations of high and low chitin within the sample. (**B**) Percentage of the total population within low and high chitin gates for cyclosporine A- and caspofungin-treated samples (*n* = 3 biological replicates). (**C**) Representative scatter plot with adjunct histograms for CFW staining and exposed β(1,3)-glucan when plotting low (blue) and high (red) chitin populations independently. (**D**) β(1,3)-glucan unmasking of cells within the low and high chitin populations following caspofungin addition to cyclosporine A-treated cells. Fluorescence intensity was normalized to median cell size for each of the two populations (***P = 0.0001 by Student’s t-test*; n* = 3 biological replicates).

Assessment of histograms from the flow cytometry data revealed that treatment with cyclosporine A and caspofungin resulted in a bimodal distribution of cells measured for chitin (Fig. 3D). However, this trend was not fully represented in the histograms for β-glucan unmasking (Fig. 3B), and thus warranted further investigation. The bimodal chitin distribution during caspofungin/calcineurin treatment revealed one population with caspofungin-like levels of chitin and another with control levels (Fig. 3D). Further gating (Fig. 4A) revealed an approximate 50/50 split in cells expressing either high (caspofungin-like) or low (WT-like) chitin content during cyclosporine A and caspofungin co-treatment (Fig. 4B). Interestingly, when assessing the unmasking levels in cells within the high and low chitin populations, we observed a significant correlation between cells with higher chitin content and increased unmasking as compared to cells with low chitin levels (Fig. 4C). However, these high and low chitin populations also correlated with cell size (Fig. S5). Comparison of unmasking within these two populations (following normalization to their respective median sizes) revealed a significant increase in β(1,3)-glucan exposure in the high chitin population, which corresponded to a 1.91-fold increase in unmasking (Fig. 4D). Thus, it appears that calcineurin mediates both unmasking and chitin deposition in response to caspofungin exposure, and β(1,3)-glucan unmasking strongly correlates with total chitin content within the cell.

### Loss of Mkc1 attenuates caspofungin-induced unmasking and chitin synthesis within the yeast cell wall

**Fig 5:**
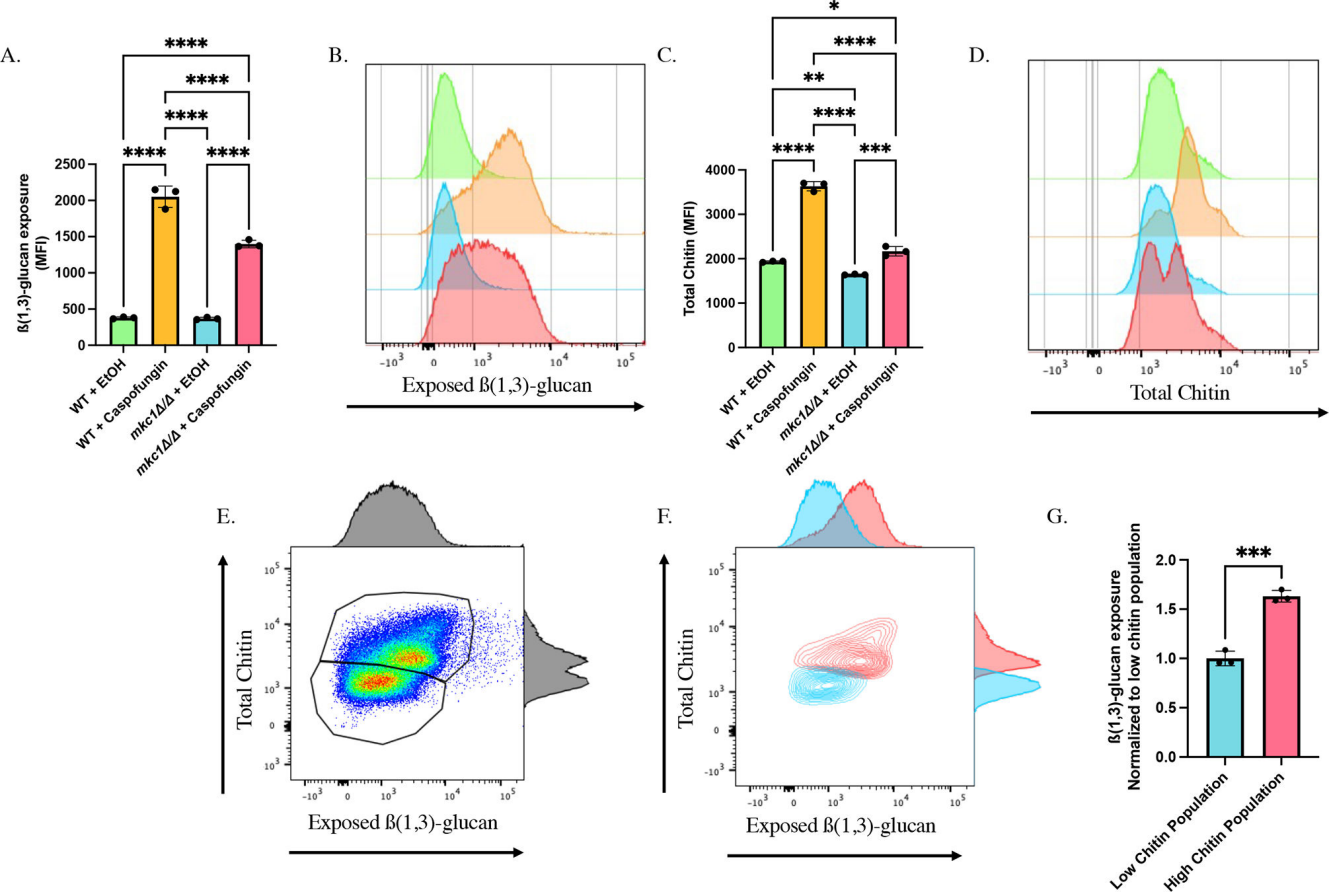
Loss of *MKC1* attenuates caspofungin-driven unmasking and chitin synthesis in yeast cells. (**A–D**) *mkc1Δ/Δ* cells grown to mid-log phase were exposed to 46.9 ng/mL of caspofungin (or EtOH solvent control) for 30 minutes, and β(1,3)-glucan exposure and total chitin levels were assessed by flow cytometry. (**A**) β(1,3)-glucan unmasking in *mkc1Δ/Δ* cells treated with sublethal concentrations of caspofungin (****P < 0.0001 by one-way ANOVA*; n* = 3 biological replicates). (**B**) Representative histogram of the impact that loss of *MKC1* has on caspofungin-induced unmasking. Colors of each sample match those shown in Fig. 5A. (**C**) CFW staining of cells treated with sublethal concentrations of caspofungin (*P < 0.01, **P < 0.01, ***P < 0.0005, ****P < 0.0001 by one-way ANOVA*; n* = 3 biological replicates). (**D**) Representative histogram of the impact that loss of *MKC1* has on caspofungin-induced chitin synthesis. Colors of each sample match those shown in 5A and C. (**E**) Representative scatter plot and adjunct histograms for CFW staining and exposed β(1,3)-glucan of *mkc1Δ/Δ* cells treated with caspofungin. Gates represent populations of high and low chitin within the sample. (**F**) Representative scatter plot with adjunct histograms for CFW staining and exposed β(1,3)-glucan when plotting low (blue) and high (red) chitin populations independently. (**G**) β(1,3)-glucan unmasking of cells within the low and high chitin populations following caspofungin addition to *mkc1Δ/Δ* cells. Fluorescence intensity was normalized to median cell size for each of the two populations (***P = 0.0003 by Student’s t-test*; n* = 3 biological replicates).

Our data show a role for calcineurin in mediating caspofungin-induced unmasking and chitin deposition within yeast cells (Fig. 1 and 2). However, loss of calcineurin only diminishes the unmasking by ~38%, suggesting that there are additional mechanisms that drive this process. In addition to calcineurin, the MAPK Mkc1 has also been implicated as a major regulator of chitin synthesis in response to caspofungin exposure (48). Furthermore, caspofungin-mediated crosstalk between both calcineurin and Mkc1 has recently been observed (49). We were therefore curious if calcineurin is interacting with Mkc1 to regulate changes in chitin levels in response to drug treatment, or if it works independently of calcineurin to mediate this phenotype. To assess this, we generated an *mkc1Δ/Δ* mutant and assessed the impact that loss of this MAPK had on caspofungin-induced unmasking and chitin synthesis. Similarly to what was observed during calcineurin inhibition (Fig. 3), *mkc1Δ/Δ* cells showed significant reductions in both unmasking (Fig. 5A and B) and chitin levels (Fig. 5C and D) after caspofungin exposure. Moreover, assessment of *mkc1Δ/Δ* cells treated with caspofungin showed the same bimodal distribution in chitin staining (Fig. 5D) that was observed in cyclosporine A- and caspofungin-treated cells (Fig. 3D). Gating on the two chitin populations in *mkc1Δ/Δ* caspofungin-treated cells again revealed a strong and significant trend in unmasking levels between high and low chitin populations within these samples (following normalization to size) (Fig. 5E through G). The observed difference between the high and low chitin populations corresponded to a 1.65-fold difference in unmasking between them (Fig. 5G). Accordingly, microscopy revealed a range of unmasking phenotypes in the *mkc1Δ/Δ* samples treated with caspofungin (Fig. S6), with a subset of cells showing reduced unmasking. Collectively, this implicates Mkc1 as a regulator of chitin synthesis and unmasking during caspofungin addition. Furthermore, the strong and persistent correlation between chitin levels and unmasking further connects chitin as a driver of β(1,3)-glucan exposure.

### Calcineurin and Mkc1 independently contribute to caspofungin-induced chitin synthesis and unmasking

**Fig 6:**
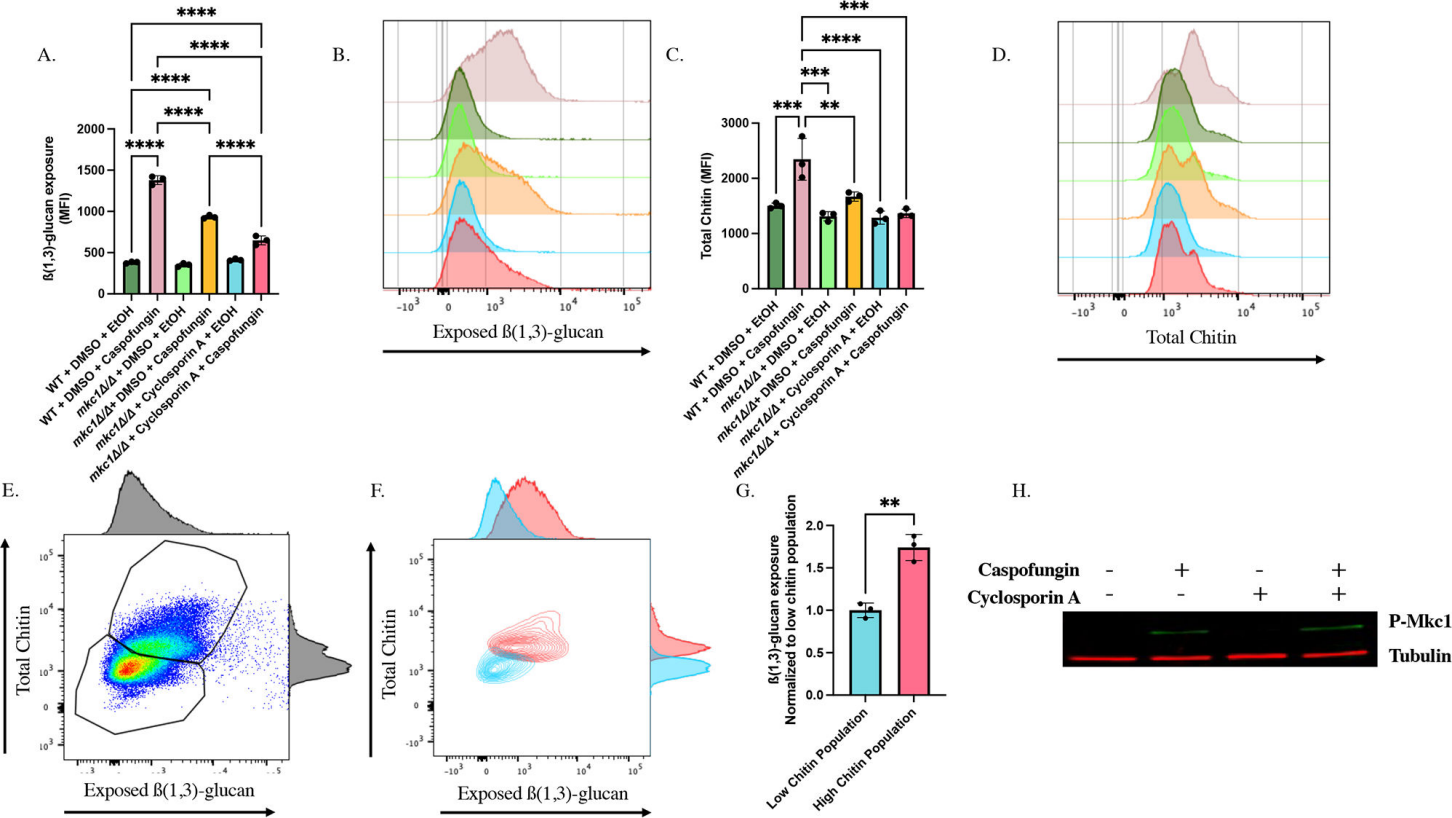
Calcineurin and Mkc1 work in parallel to mediate caspofungin-induced chitin synthesis and unmasking. (**A–D**) *mkc1Δ/Δ* cells grown to mid-log phase in either the presence of 100 µg/mL of cyclosporine A, or the appropriate solvent control, were exposed to 46.9 ng/mL of caspofungin (or EtOH solvent control) for 30 minutes. β(1,3)-glucan exposure and total chitin levels were then assessed by flow cytometry. (**A**) β(1,3)-glucan exposure of *mkc1Δ/Δ* cells treated with cyclosporine A and caspofungin (****P < 0.0001*; n* = 3 biological replicates). (**B**) Representative histogram of the impact that calcineurin inhibition has on caspofungin-induced unmasking in an *mkc1Δ/Δ* background. Colors of each sample match those shown in Fig. 6A. (**C**) CFW staining of *mkc1Δ/Δ* cells treated with cyclosporine A (calcineurin-inhibited) and caspofungin (**P < 0.01, ***P < 0.001,****P < 0.0001, by one-way ANOVA*; n* = 3 biological replicates). (**D**) Representative histogram of the impact that calcineurin inhibition has on caspofungin-induced chitin synthesis in an *mkc1Δ/Δ* background. Colors of each sample match those shown in Fig. 6A and C. (**E**) Representative scatter plot and adjunct histograms for CFW staining and exposed β(1,3)-glucan of *mkc1Δ/Δ* cells treated with cyclosporine A and caspofungin. Gates represent populations of high and low chitin within the sample. (**F**) Representative scatter plot with adjunct histograms for CFW staining and exposed β(1,3)-glucan when plotting low (blue) and high (red) chitin populations independently. (**G**) β(1,3)-glucan unmasking of cells within the low and high chitin populations following caspofungin exposure and cyclosporine A treatment of *mkc1Δ/Δ* cells. Fluorescence intensity was normalized to median cell size for each of the two populations (**P = 0.0019 by Student’s t-test*; n* = 3 biological replicates). (**H**) Western blot analysis of Mkc1 activation in response to cyclosporine A and caspofungin exposure. Cells were grown to mid-log phase in the presence of 100 µg/mL of cyclosporine A or an appropriate volume of the DMSO solvent control, and were exposed to 46.9 ng/mL of caspofungin (or EtOH solvent control) for 30 minutes, and proteins were harvested for western blot analysis. Membranes were blotted using an anti-P44/42 antibody for phosphorylated (active) Mkc1 detection. An anti-tubulin antibody was used as a loading control.

**Fig 7:**
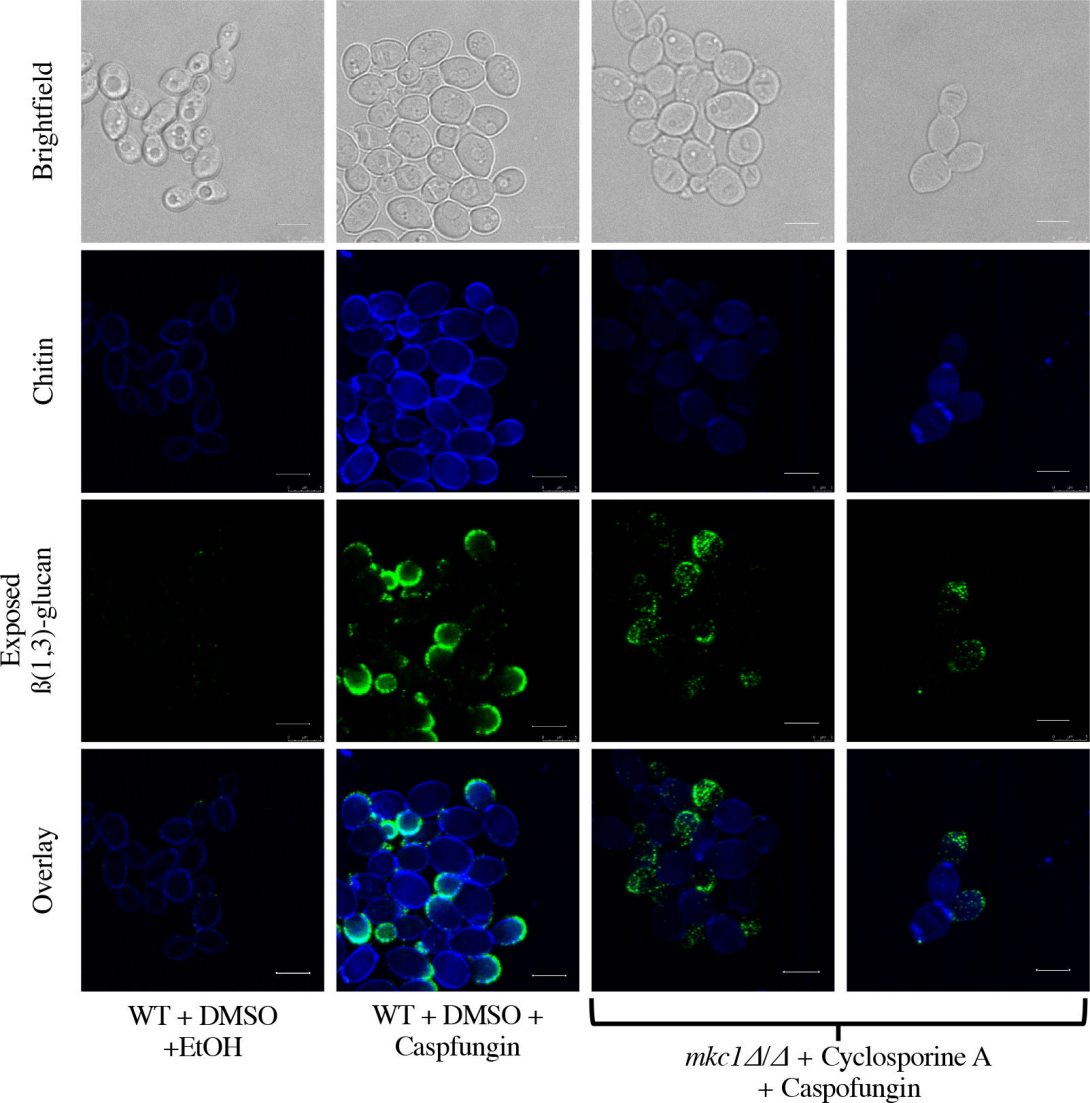
Inhibition of calcineurin in an *mkc1Δ/Δ* mutant attenuates caspofungin-induced unmasking and chitin synthesis. Representative microscopy images of calcineurin-inhibited *mkc1Δ/Δ* cells exposed to caspofungin or an ethanol solvent control. *mkc1Δ/Δ* cells grown to mid-log phase in either the presence of 100 µg/mL of cyclosporine A, or the appropriate solvent control, were exposed to 46.9 ng/mL of caspofungin (or EtOH solvent control) for 30 minutes, and β(1,3)-glucan exposure and total chitin levels were assessed (scale bar indicates 5 µm).

Given the similarities in unmasking and chitin trends between calcineurin-inhibited and *mkc1Δ/Δ* cells treated with caspofungin (Fig. 3 and 5), we hypothesized that calcineurin induces changes in unmasking and chitin *via* Mkc1 activation. To assess the linearity of this pathway, we treated *mkc1Δ/Δ* cells with cyclosporine A prior to caspofungin addition and measured the levels of β(1,3)-glucan exposure and chitin within the cell wall *via* flow cytometry. Inhibition of calcineurin in an *mkc1Δ/Δ* mutant treated with caspofungin further reduced both unmasking and chitin levels compared to the *mkc1Δ/Δ* mutant treated with caspofungin alone (Fig. 6A through D), suggesting independent roles for these pathways. Additionally, microscopy revealed an increase in cells displaying reduced β(1,3)-glucan exposure and significantly less CFW staining (Fig. 7).

As can be seen from the histogram of chitin levels (Fig. 6D), there was still a subpopulation of cells inhibited for both pathways that exhibited high levels of chitin during caspofungin treatment, but it was a smaller peak within the whole population than when either pathway alone was inhibited (Fig. 3D and Fig. 5D). Consistently with inhibition of either pathway alone, the high chitin population exhibited higher levels of unmasking (Fig. 6F and G).

In further support that calcineurin does not act *via* Mkc1 activation, western blot analysis did not show a difference in active (phosphorylated) Mck1 levels in WT cells treated with caspofungin alone when compared to cells treated with both cyclosporine A and caspofungin (Fig. 6H). Thus, although Mkc1 and calcineurin both impact chitin synthesis and unmasking in response to caspofungin exposure, they appear to work in parallel with each other to induce these changes. Yet, the persistent observation that chitin levels correlate with increased β(1,3)-glucan exposure further implicates chitin as a driver for caspofungin-induced unmasking.

### Chitin synthase 3 levels increase during caspofungin treatment, but do not correlate with changes in chitin content

**Fig 8:**
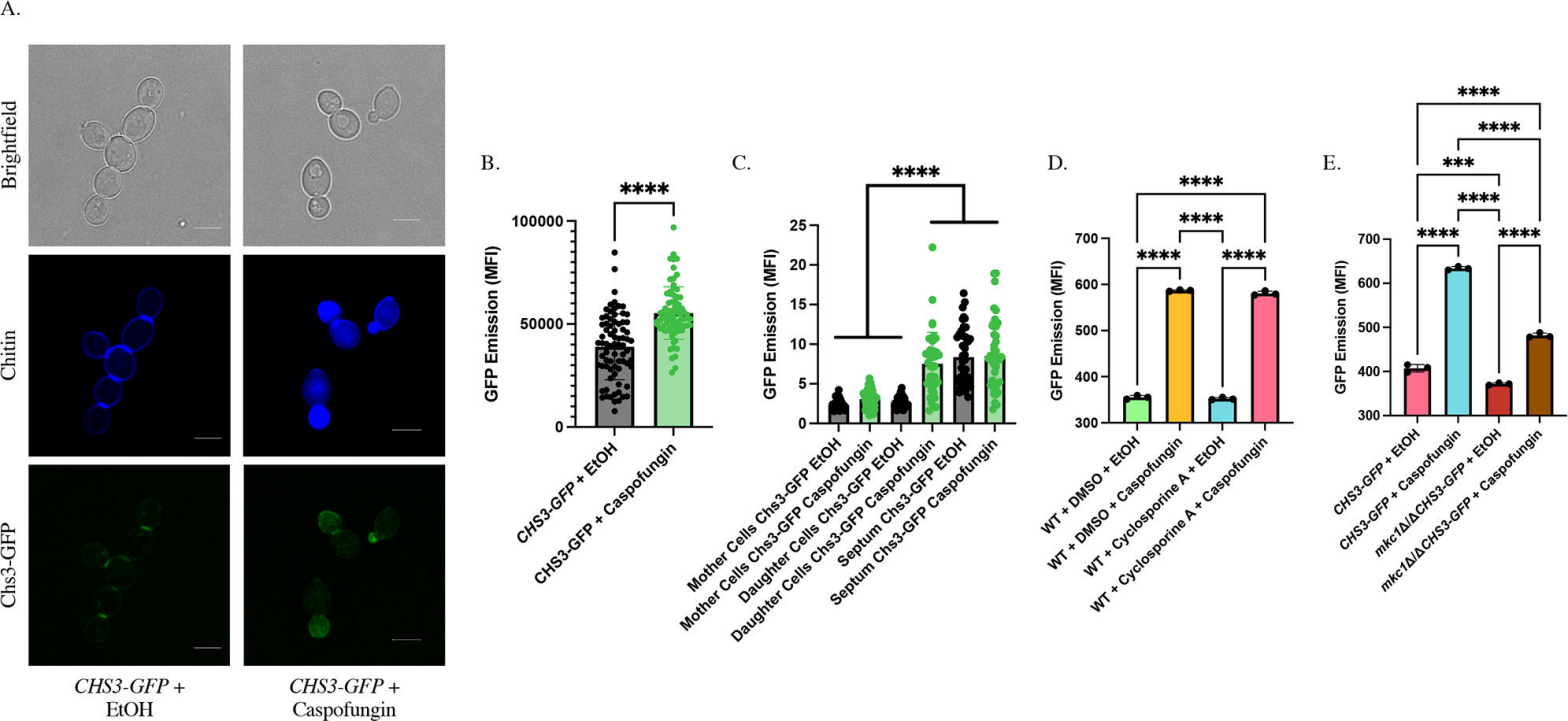
Mkc1 regulates *CHS3* levels in yeast cells in response to caspofungin. *CHS3-GFP* WT cells grown to mid-log phase were exposed to 46.9 ng/mL of caspofungin for 30 minutes, stained with CFW, and the corresponding GFP and CFW emission was assessed *via* microscopy and flow cytometry. (A) Representative images of *CHS3-GFP* cells exposed to caspofungin or an ethanol solvent control (scale bar indicates 5 µm). (B) Quantification of GFP emission in ethanol-treated and caspofungin-treated cells (*n* = 75 cells per treatment; ****P < 0.0001 by Mann–Whitney test). (C) Chs3-GFP quantifications of microscopy images in mother cells, daughter cells, and septa of budding yeast (*n* = 42 budding cells analyzed per treatment; ****P < 0.0001 by one-way ANOVA). (D) GFP emission of *CHS3-GFP* WT cells treated with cyclosporine A and caspofungin (****P < 0.0001 by one-way ANOVA*; n* = 3 biological replicates). (E) GFP emission of *mkc1Δ/Δ CHS3-GFP* cells treated with caspofungin (***P < 0.0005, ****P < 0.0001 by one-way ANOVA*; n* = 3 biological replicates).

Given the observation that changes in chitin content repeatedly correlate with changes in β(1,3)-glucan exposure, we next wanted to assess how these alterations may be occurring upon caspofungin treatment. *C. albicans* has four major chitin synthase enzymes (55), and altered expression of these genes is capable of dictating the levels of chitin within the cell wall at various stages of growth or in response to exogenous stimuli (56). Chitin synthase 3 (Chs3) is the major chitin synthase enzyme responsible for chitin deposition within the cell wall (57, 58), and it has been identified as the major chitin synthase enzyme responsible for increasing chitin content during caspofungin exposure (48). Therefore, we hypothesized that Chs3 plays a role in caspofungin-induced unmasking. To test this, we genomically integrated a *CHS3* gene with a C-terminal green fluorescent protein tag (*CHS3-GFP*) at its native locus and assessed how caspofungin exposure impacts Chs3-GFP protein levels and localization. We found that WT cells treated with an ethanol solvent control show low levels of Chs3-GFP, but that Chs3-GFP fluorescence significantly increases upon caspofungin exposure (Fig. 8A). Quantification of the fluorescent intensity of the cells in these microscopy images revealed a 1.42-fold increase in fluorescence following caspofungin treatment (Fig. 8B). Additionally, quantification of Chs3-GFP localization showed a predominant localization to the septa during growth in an ethanol solvent control, but robust localization to daughter cells during caspofungin treatment (Fig. 8C). This fits well with the observation that increased chitin deposits predominantly co-localize with unmasking in budding daughter cells (Fig. 1) and supports the idea that Chs3 may mediate these changes.

We next wished to assess if calcineurin and/or Mkc1 regulate Chs3 levels in response to caspofungin. To assess the impact that calcineurin has on *CHS3* expression, we pretreated the *CHS3-GFP* mutant with cyclosporine A prior to caspofungin exposure. Overall, flow cytometry did not reveal a change in GFP fluorescence between the cyclosporine A- and caspofungin-treated samples when compared to caspofungin only-treated cells (Fig. 8D), suggesting that calcineurin does not directly regulate caspofungin-induced Chs3 expression. However, an *mkc1Δ/ΔCHS3-GFP* double mutant did show a significant reduction in fluorescent intensity when compared to the *CHS3-GFP* mutant in a WT background (Fig. 8E). Therefore, it appears that Mkc1, but not calcineurin, is an important regulator of *CHS3* expression in response to caspofungin. Gating on the high and low chitin populations within the *mkc1Δ/ΔCHS3-GFP* strain also revealed a similar trend as was seen for unmasking in these same conditions (Fig. S7A and B). However, following size normalization between the two chitin populations, no significant difference was observed in Chs3-GFP levels between them (Fig. S7C). Thus, although caspofungin significantly increases Chs3 levels in an Mkc1-dependent manner, this does not clearly correlate with changes in chitin content and unmasking following loss of *MKC1*.

### Caspofungin-induced unmasking relies on chemical inhibition of *FKS1*

Our data highlight a tight relationship between total chitin content and unmasking during caspofungin exposure. However, it is unknown if unmasking is induced by direct inhibition of the β(1,3)-glucan synthase Fks1. To test this, we utilized two caspofungin-resistant isolates that harbor an S645P mutation in the Fks1 protein that impairs caspofungin inhibition of this enzyme (42). Staining for β(1,3)-glucan exposure and chitin revealed a baseline increase in both unmasking and total chitin within these isolates when compared to the SC5314-derived *LEU2/leu2∆* WT control (Fig. S8A and B). This is in agreement with studies assessing other caspofungin-resistant isolates, where increased chitin is commonly observed (51 - 53). However, treatment with caspofungin did not increase the unmasking in either of the resistant isolates (Fig. S8C), suggesting that chemical inhibition of the Fks1 protein is indeed necessary to induce unmasking following caspofungin addition.

## DISCUSSION

We have previously shown that calcineurin inhibition attenuates caspofungin-induced unmasking in *C. albicans*. However, the mechanism by which calcineurin impacts β(1,3)-glucan exposure is unclear. In this report, we show that pockets of β-glucan unmasking strongly co-localize with areas of increased chitin deposition in response to caspofungin (Fig. 1C), and that pharmacological inhibition of chitin synthesis *via* nikkomycin Z attenuates this phenotype (Fig. 2). Additionally, both calcineurin (Fig. 3) and the MAPK Mkc1 (Fig. 5) independently regulate unmasking and chitin synthesis in response to caspofungin. Interestingly, caspofungin-treated cells that are inhibited for calcineurin or have *MKC1* deleted displayed a bimodal distribution of total chitin content that strongly correlated with unmasking levels (Fig. 3 and 5), further implicating chitin synthesis as a driver for unmasking. Our data also indicate that there are chitin-independent contributions to unmasking. The loss of calcineurin and Mkc1 activity simultaneously or inhibition of chitin synthases by nikkomycin Z during caspofungin exposure reduces chitin levels to those of untreated controls, yet unmasking of β(1,3)-glucan is reduced by only about 50% (Fig. 2 and 6). Microscopy analysis also supports this, as cells displaying minimal CFW staining still showed unmasking, although reduced, in both experimental conditions (Fig. 2 and 7).

**Fig 9:**
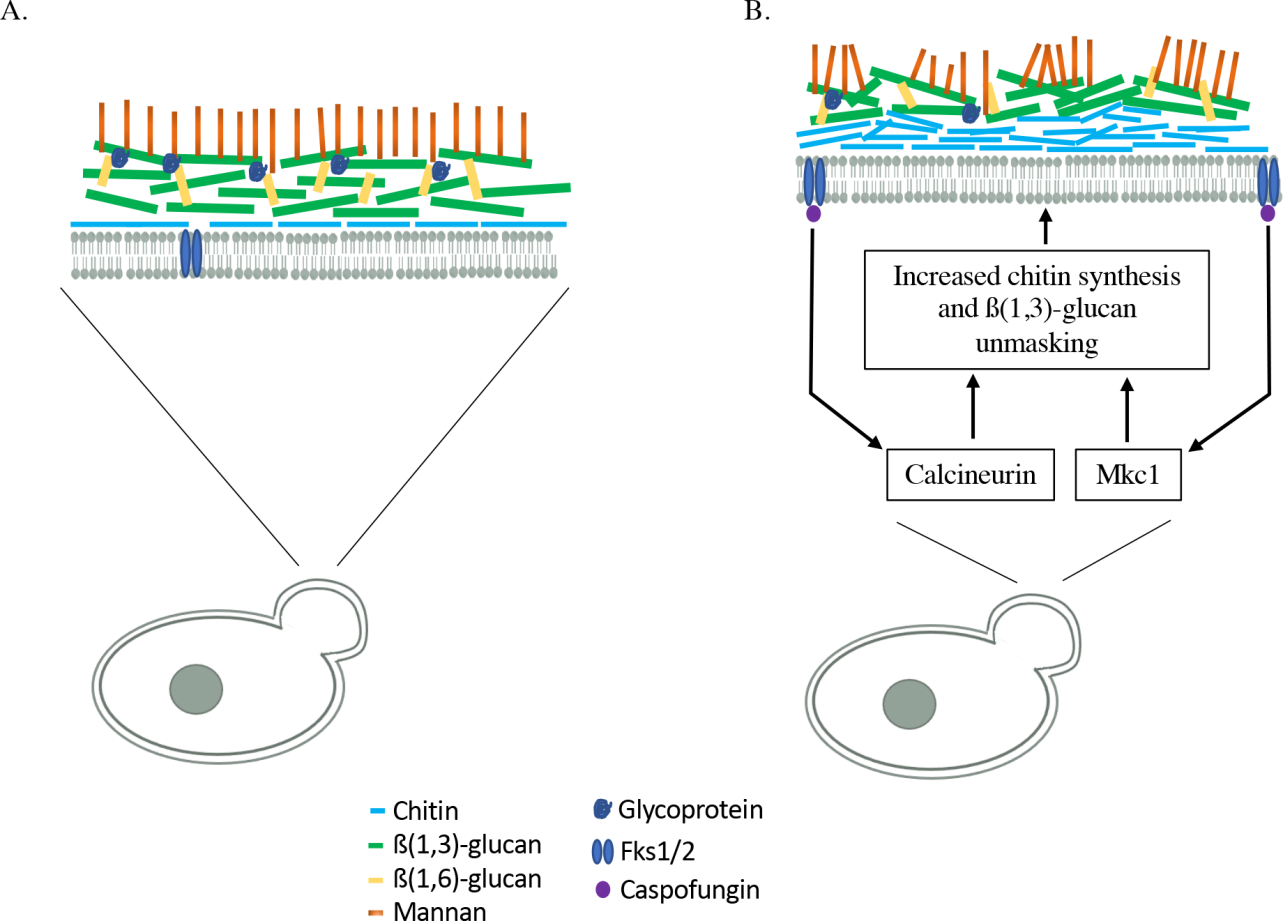
Proposed model for caspofungin-induced unmasking in growing yeast cells. (**A**) Cell wall organization of a budding daughter cell in exponentially growing cells. (**B**) The cell wall organization of budding daughter cells following exposure to caspofungin. Inhibition of the Fks enzymes by caspofungin leads to activation of both Mkc1 and calcineurin. In turn, both pathways work independently to regulate expression and proper localization of chitin synthase enzymes to the pole of budding daughter cells. This in turn increases chitin synthesis at this location, leading to a disruption of the cell wall architecture and ultimate exposure of central β(1,3)-glucan moieties to the surrounding environment.

Our data describe mechanisms that account for the strong correlations between foci of exposed β(1,3)-glucan and increased chitin deposition during caspofungin exposure. This adds to the few mechanisms that have been described that correlate chitin content and unmasking in response to genetic manipulation or exogenous stressors. For example, increased chitin content in hyphae corresponds to areas of unmasking induced by NETosis (59), and unmasking in hyphal cells of clinical vaginal isolates co-localize with areas of increased chitin (60). Furthermore, strains disrupted for the phosphatidylserine synthase *CHO1* gene or overexpressing a hyperactive allele of the MAP3K *STE11* display increases in chitin and unmasking (32, 33, 38). Collectively, these observations support a model in which increased chitin deposition disrupts fungal cell wall architecture resulting in β(1,3)-glucan exposure (Fig. 9). Indeed, compensatory mechanisms driving robust realignments in cell wall architecture have been noted by others (61), and evidence exists for alternative unmasking mechanisms in addition to increased chitin synthesis. For example, alterations in N-glycosylation and glycophosphatidylinositol-anchored protein synthesis increase β-glucan exposure by disrupting the outer mannose layer of the cell wall (62 - 64). Additionally, expression of the exoglucanase *XOG1*, *via* exogenous lactate exposure, reduces unmasking *via* “epitope shaving” of exposed glucan fibrils (24). Since unmasking still occurred at reduced levels in calcineurin-inhibited *mkc1Δ/Δ* cells treated with caspofungin, where chitin levels are comparable to the untreated WT control (Fig. 6), some of these other mechanisms may be involved as well.

This report implicates calcineurin and Mkc1 as drivers for inducing increased chitin synthesis and unmasking in response to caspofungin. This observation supports previous studies showing a major regulatory role for these pathways in inducing changes in chitin content during caspofungin treatment (48). However, we have found that these pathways work independently of each other in an additive fashion (Fig. 6), which differs from previously published results that show crosstalk between calcineurin and Mkc1 in response to caspofungin. This previous work reported a regulatory role for calcineurin in reducing Mkc1-mediated chitin synthesis during exposure to high concentrations of caspofungin (49). However, the drug concentrations used in those studies were ~40-times greater than the concentrations used in this study and dealt with cells experiencing the “paradoxical growth effect,” in which *C. albicans* cells can grow and persist at drug treatments well above the MIC (65). We believe that these discrepancies in assay conditions account for the differences observed in calcineurin and Mkc1 crosstalk. However, it is still entirely possible that each of these pathways play a redundant and compensatory role when one of them is perturbed, which still highlights the interconnected nature of signal transduction pathways during caspofungin treatment.

Although Mkc1 and calcineurin have both been implicated in driving chitin synthesis and unmasking in response to caspofungin, the exact mechanism through which drug-induced cell wall changes are made remains poorly understood. Microscopy revealed increased areas of chitin deposition at the poles of daughter yeast cells that strongly co-localized with pockets of unmasking (Fig. 1). Chitin is the last polymer deposited within the cell wall of budding daughter cells in *Saccharomyces cerevisiae* (66, 67), and an increase in compensatory chitin deposition at these areas makes sense, as these are likely the most structurally unstable points within budding cells. However, how these weak areas are sensed and how chitin is directed to these foci are still unknown. Our data show that Chs3 levels are increased in daughter cells following drug treatment (Fig. 8) and its expression is, in part, Mkc1 dependent. However, no correlation was observed between Chs3-GFP levels and the high and low chitin populations in caspofungin-treated *mkc1Δ/Δ* cells. This suggests that Chs3 likely plays a role in mediating chitin synthesis in budding daughter cells in response to caspofungin, but that alternative chitin synthases may be mediating the changes in unmasking. Additionally, the transcription factors mediating the observed changes in chitin synthesis and the signals that direct precise post-translational localization of the chitin synthase enzymes to the membrane following caspofungin exposure remain elusive. A link between altered phosphatidylinositol 4,5-biphosphate [PI(4,5)P_2_], an important precursor of secondary messengers and septin localization, has been observed during caspofungin treatment (68, 69). Interestingly, these altered foci also co-localize with areas of increased chitin. In *S. cerevisiae*, PI(4,5)P_2_ is a regulator of Rho1 activation (70), the upstream GTPase of Mkc1 and a regulator of the Fks enzymes (71 - 74). It may be possible that caspofungin exposure stimulates Mkc1 *via* this PI(4,5)P_2_-Rho1 interaction in *C. albicans*, and may help direct the appropriate enzymes to mediate chitin synthesis at specific foci within the cell wall. However, further analysis on these processes is needed.

## MATERIALS AND METHODS

### Strain construction

Details on growth media, strains, and plasmid construction can be found in Text S1.

### Immunofluorescent staining, flow cytometry, and microscopy analysis

To quantify caspofungin-induced β(1,3)-glucan exposure and chitin synthesis, 5 mL overnight cultures of *C. albicans* strains in YPD (10% yeast extract, 20% peptone, 20% dextrose) medium were started the day prior to staining, and were incubated shaking overnight at 225 rpm at 30°C. The following morning, strains were back-diluted to an OD_600_ of 0.1 in YPD. Cells were grown for 3 hours, shaking at 225 rpm at 30°C. To assess the impact of calcineurin on caspofungin-induced unmasking and chitin deposition, 100 ug/mL of cyclosporine A or the DMSO solvent control was added to the media prior to back-dilution in the morning. In either control or cyclosporine A-treated samples, after 3 hours of incubation, 46.9 ng/mL of caspofungin, or ethanol solvent control, was added to each tube, and strains were incubated at 225 rpm at 30°C for an additional 30 minutes. For assays involving nikkomycin Z, either 16 µg/mL or 6.5 µg/mL of nikkomycin Z (solubilized in water) was added along with caspofungin, and incubated at 225 rpm at 30°C for an additional 30 minutes.

Following incubation, 1 mL of each culture was removed to assess β(1,3)-glucan exposure and total chitin content as previously described (36, 38). For flow cytometry, an anti-β(1,3)-glucan primary antibody (Biosupplies Australia, Bundoora, Australia) and a goat anti-mouse secondary antibody conjugated to R-phycoerythrin (Jackson Immuno Research, West Grove, PA, USA) at a 1:300 dilution were used, while a rabbit anti-mouse IgG secondary antibody conjugated to Alexa Fluor 488 (Jackson Immuno Research) was used at a 1:300 dilution for microscopy. To simultaneously assess total chitin levels, samples were washed once with 500 µL of PBS following incubation with the secondary antibody used for β-glucan staining, and then were incubated with 500 µL of 10 µg/mL CFW (Fisher Scientific, Waltham, MA, USA) in water for 5 minutes while rocking at room temperature. Samples were subsequently washed three more times. For flow cytometry, samples were resuspended in 500 µL of FACS (fluorescence-activated cell sorting) buffer (1× PBS + 0.01% sodium azide) for analysis. Samples prepared for microscopy were not fixed but were resuspended in 300 µL of water prior to visualization on the confocal microscope.

For all conditions stained for flow cytometry, three biological replicates consisting of 100,000 recorded events each were measured. Visualization and data analyses were performed in FlowJo (Becton, Dickinson and Company, Franklin Lakes, NJ, USA). Statistical significance between median fluorescent intensities of each experimental group was determined by a Student’s t-test, Mann–Whitney test (for samples with a non-Gaussian distribution, as determined by a D’Agostino-Pearson omnibus normality test), or a one-way analysis of variance (ANOVA) with Tukey’s post hoc analysis (GraphPad Prism, v7.0c software). For analysis of β(1,3)-glucan exposure between high and low chitin populations within a single sample, the median relative fluorescent intensities were normalized to the median forward scatter area, representing relative size, for each respective population prior to data visualization and statistical analysis.

For microscopy, ≥3 biological replicates for all conditions were analyzed on a Leica SP8 White Light Laser Confocal Microscope. Fluorescent intensities were analyzed *via* ImageJ (National Institute of Health, Bethesda, MD, USA). To measure mother cell, daughter cell, and septal chitin content and unmasking, daughter and mother cells were differentiated by size and the presence of bud scars, and the lateral cell wall and septal staining intensities were recorded. In total, four biological replicates from 2 separate days were assessed with >4 fields of view per sample, equaling 38 budding cells quantified in total. For GFP measurements, 75 individual cells were quantified from three biological replicates for total fluorescence measurement, and 42 budding cells were assessed to quantify intracellular localization. Once counts were obtained from all analyses, outliers were determined *via* an outlier test and distribution normality was assessed with the use of a D’Agostino-Pearson omnibus normality test (GraphPad Prism, v7.0c software). Statistical significance was determined *via* a Student’s t-test, a Mann–Whitney test, a one-way ANOVA with Tukey’s post hoc analysis, or a non-parametric Kruskal–Wallis test (GraphPad Prism, v7.0c software).

Hyphal cells were assessed by back-diluting stationary cells to an OD_600_ of 0.1 in PBS. Following dilution, 200 µL of cells was transferred to a fresh Eppendorf tube, centrifuged, and resuspended in 1 mL of RPMI (Roswell Park Memorial Institute) medium, and 200 µL of this suspension was added to the wells of an 8-well Permanox slide. Cells were incubated at 37°C for 3 hours, and then the media was replaced with fresh RPMI containing either 46.9 ng/mL of caspofungin or an ethanol solvent control, and left to incubate at 37°C for 30 minutes. Staining was then performed as previously described and cells were analyzed *via* microscopy.

### Spot dilution assays

To assess the viability of cells following drug exposure, strains were grown in the presence of cyclosporine A and caspofungin as described above. Following drug treatment, samples were washed three times with 500 µL of PBS and back-diluted to an OD_600_ of 0.1 in PBS, and four subsequent 1:10 dilutions were performed. Three microliters of each dilution was then spotted onto a fresh YPD plate. Plates were then incubated for 24 hours at 30°C, and images were taken the following day.

### Western blot analysis of phosphorylated Mkc1

Activated (phosphorylated) levels of Mkc1 were assessed as previously described (37)

## SUPPLEMENTAL MATERIAL

Supplemental Figures mbio.00074-23-s0001.pdf Figures S1-S8 plus legends.

Table S1 mbio.00074-23-s0002.docx Strains used in this study.

Table S2 mbio.00074-23-s0003.docx Primers used in this study.

Table S3 mbio.00074-23-s0004.docx Plasmids used in this study.

Text S1 mbio.00074-23-s0005.docx Growth conditions and cloning methods used in this study.

ASM does not own the copyrights to Supplemental Material that may be linked to, or accessed through, an article. The authors have granted ASM a non-exclusive, world-wide license to publish the Supplemental Material files. Please contact the corresponding author directly for reuse.
