# Supplementary material for: Caspofungin-induced β(1,3)-glucan exposure in Candida albicans is driven by increased chitin levels
Source: mBio. 2023 Jun 28;14(4):e00074-23. doi: 10.1128/mbio.00074-23 (PMC10470516; doi:10.1128/mbio.00074-23)
Supplement: Supplemental Figures — Figures S1-S8 plus legends. [file mbio.00074-23-s0001.pdf]

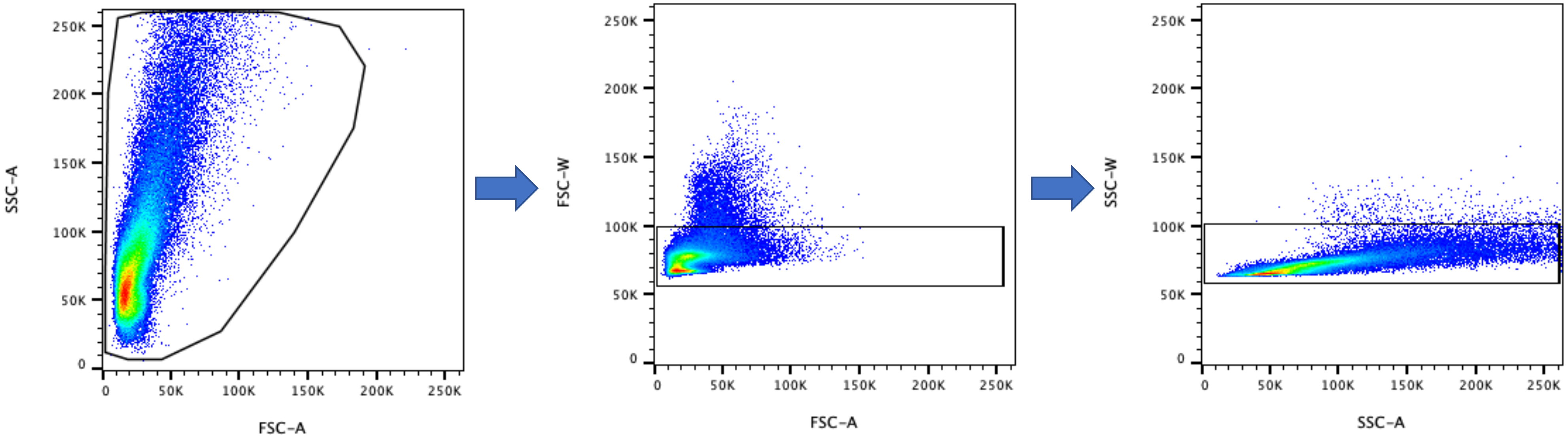

SSC-A
250K
200K
150K
100K
50K
0
0
50K
100K
150K
200K
250K
FSC-A
FSC-W
250K
200K
150K
100K
50K
0
0
50K
100K
150K
200K
250K
FSC-A
SSC-W
250K
200K
150K
100K
50K
0
0
50K
100K
150K
200K
250K
SSC-A

Fig S1: Gating strategy for single yeast cells. Gating on FSC-A vs SSC-A removes extracellular debris found in the flow cytometer. Further gating on FSC-A vs FSC-W is then used to remove larger cellular aggregates running through the machine, and is further refined via gating in SSC-A vs SSC-W. (FSC = Forward Scatter, SSC = Side Scatter, A = Area, W = Width).

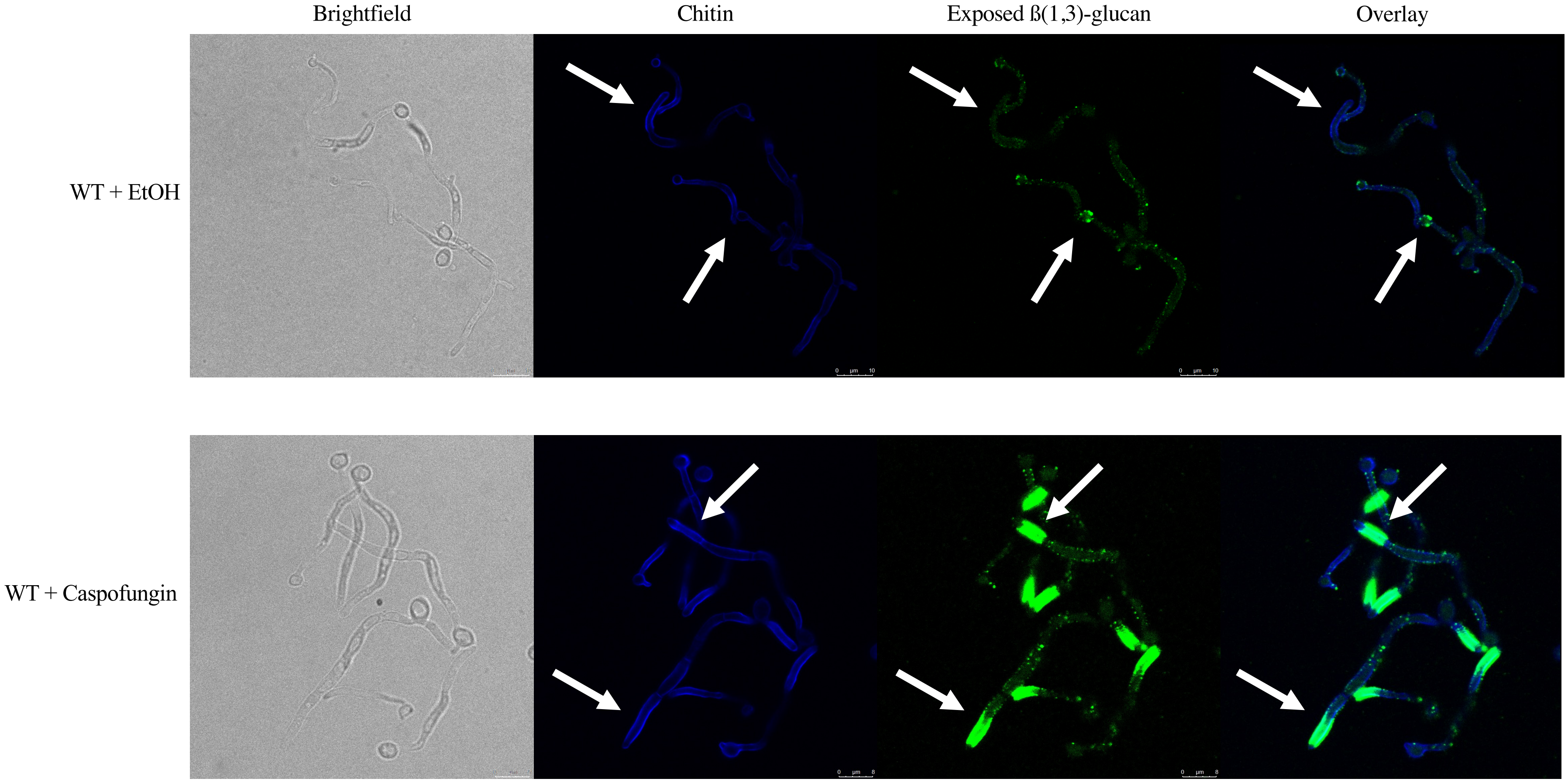

Brightfield
Chitin
Exposed ß(1,3)-glucan
Overlay
WT + EtOH
WT + Caspofungin

Fig. S2: Increased chitin synthesis and unmasking occur along the lateral cell wall near the hyphal tip.

Hyphal cells were grown for 3 hours, exposed to 46.9 ng/ml of caspofungin for 30 minutes, and then ß(1,3)-glucan exposure and total chitin were stained for microscopy. White arrows highlight examples of unmasking across images. (Scale bar indicates 10 µm).

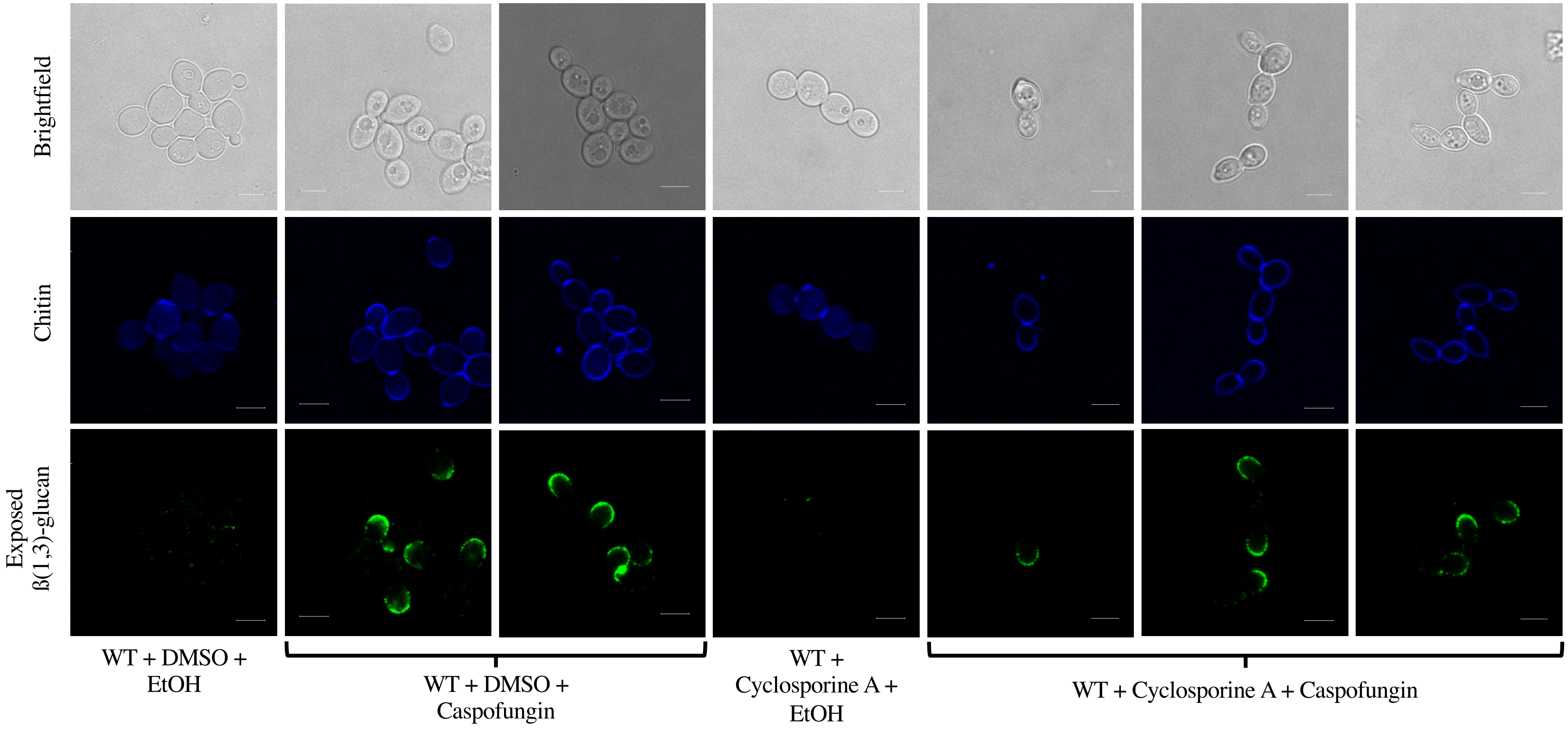
Brightfield
Chitin
Exposed
ß(1,3)-glucan
WT + DMSO +
EtOH
WT + DMSO +
Caspofungin
WT +
Cyclosporine A +
EtOH
WT + Cyclosporine A + Caspofungin

Fig. S3: Calcineurin inhibition attenuates caspofungin-induced unmasking and chitin synthesis in yeast cells.

Representative microscopy images of calcineurin inhibited cells exposed to caspofungin or an ethanol solvent control. Wild-type cells were grown to mid-log phase in either the presence of 100µg/ml of cyclosporine A, or the appropriate solvent control and then exposed to 46.9 ng/ml of caspofungin (or EtOH solvent control) for 30 minutes. ß(1,3)-glucan exposure and total chitin levels were assessed via microscopy. (Scale bar indicates 5 µm).

OD_{600} 0.1 10^{-1} 10^{-2} 10^{-3} 10^{-4}

WT + DMSO + EtOH

WT + DMSO + Caspofungin

WT + Cyclosporine A + EtOH

WT + Cyclosporine A + Caspofungin

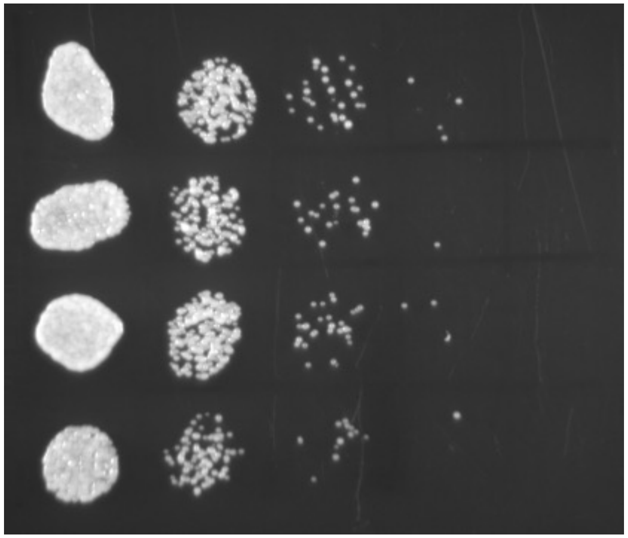

Fig S4: Calcineurin inhibition does not impact cell survival following caspofungin exposure.

Wild-type cells grown to mid-log phase in either the presence of 100µg/ml of cyclosporine A, or an appropriate volume of the DMSO solvent control, were exposed to 46.9ng/ml of caspofungin (or EtOH solvent control) for 30 minutes. Cells were then washed, back-diluted to an OD_{600} of 0.1, and serially diluted to assess viability via a spot dilution assay onto YPD.

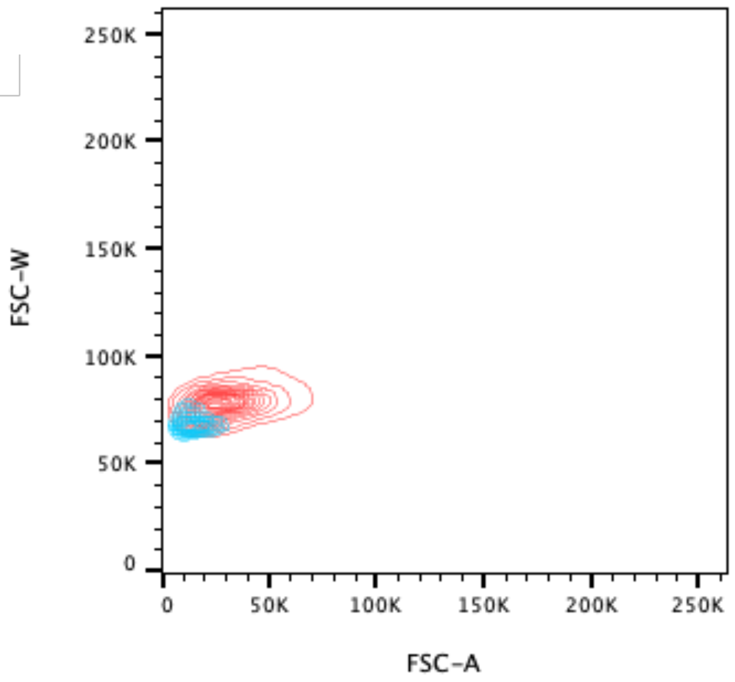

FSC-W
FSC-A
250K
200K
150K
100K
50K
0
0
50K
100K
150K
200K
250K

Fig S5: High and low chitin populations in calcineurin-inhibited cells exposed to caspofungin show size differences.
Representative contour plot of FSC-A and FSC-W of low (blue) and high (red) chitin populations in cells treated with both cyclosporine A and caspofungin.

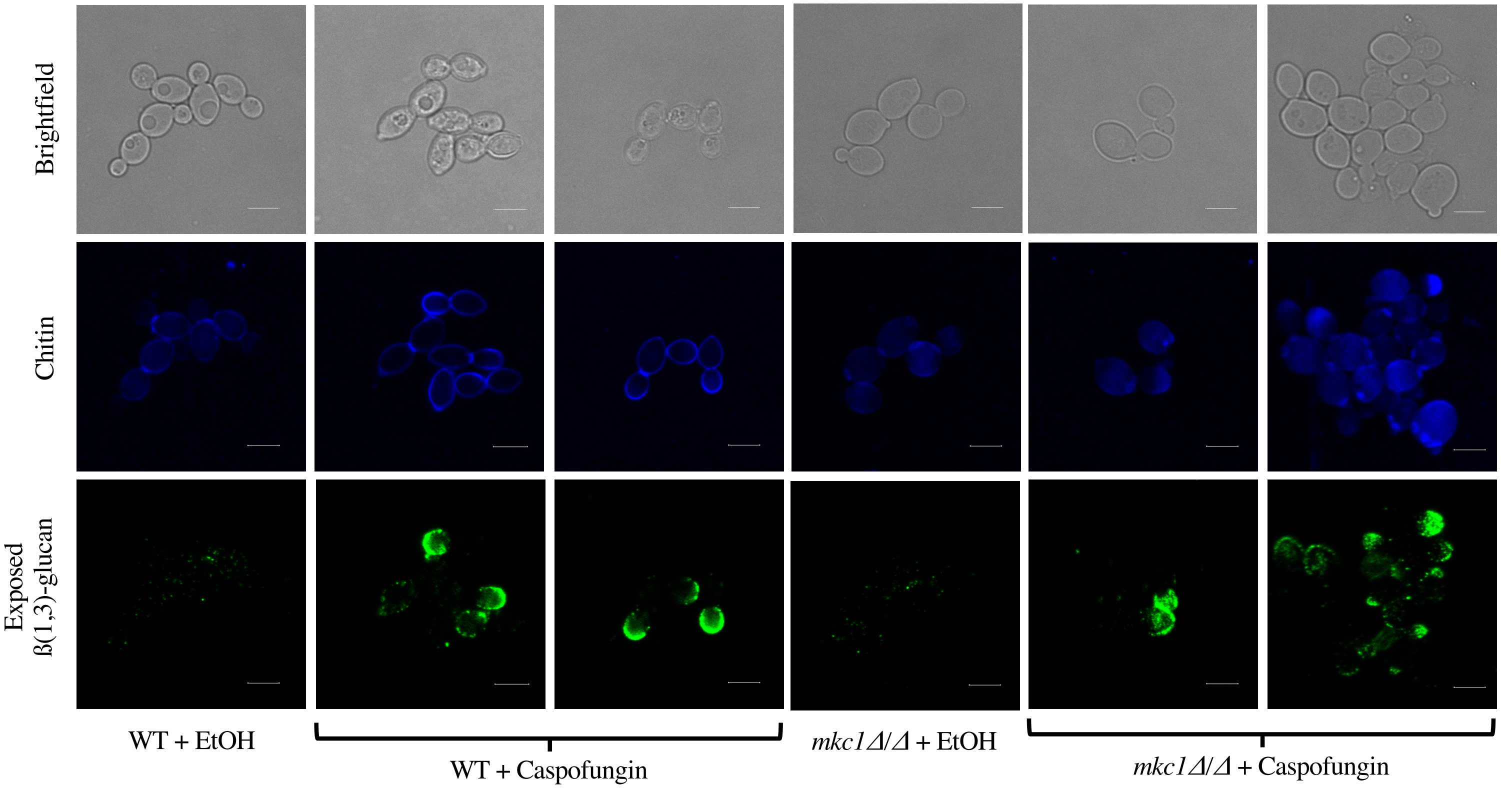

Brightfield
Chitin
Exposed β(1,3)-glucan
WT + EtOH
WT + Caspofungin
mkc1Δ/Δ + EtOH
mkc1Δ/Δ + Caspofungin

Fig. S6: Loss of *MKC1* attenuates caspofungin-induced unmasking and chitin synthesis in yeast cells.

Representative microscopy images of *mkc1Δ/Δ* cells exposed to caspofungin or an ethanol solvent control. *mkc1Δ/Δ* cells were grown to mid-log phase and then exposed to 46.9ng/ml of caspofungin (or EtOH solvent control) for 30 minutes. ß(1,3)-glucan exposure and total chitin levels were assessed via microscopy. (Scale bar indicates 5 µm).

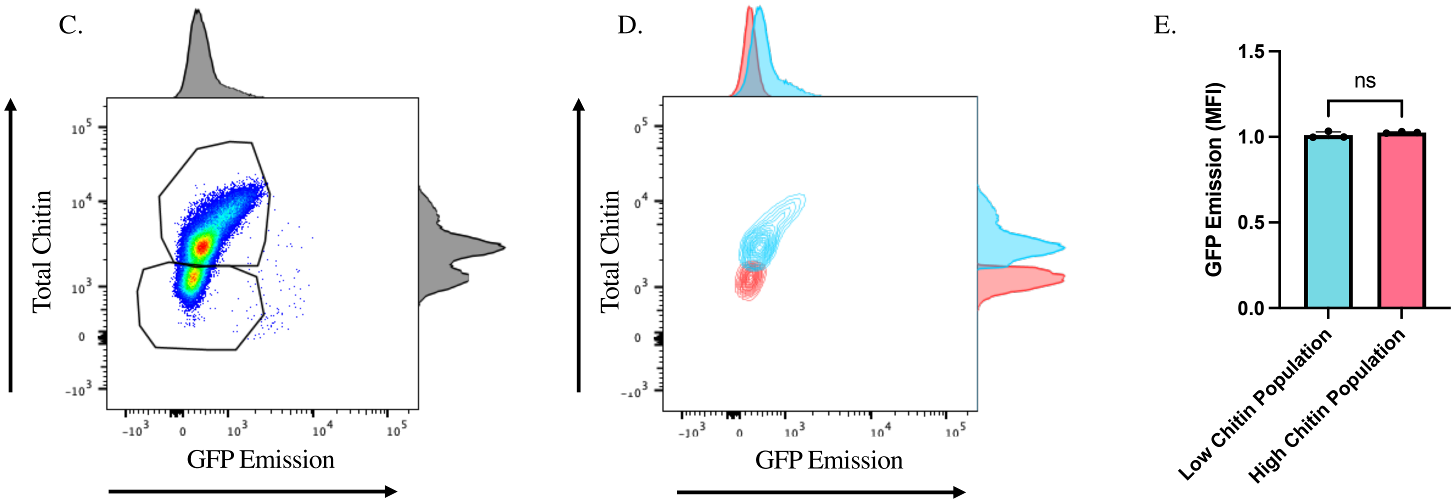

C.
Total Chitin
GFP Emission
D.
Total Chitin
GFP Emission
E.
GFP Emission (MFI)
ns
1.5
1.0
0.5
0.0
Low Chitin Population
High Chitin Population

Fig. S7: Chs3 levels do not correlate with high and low chitin populations in *mkc1Δ/Δ CHS3-GFP* yeast cells treated with caspofungin.

*mkc1*Δ/Δ *CHS3-GFP* cells grown to mid-log phase in either the presence of 100 μg/ml of cyclosporine A, or an appropriate volume of the DMSO solvent control, were exposed to 46.9 ng/ml of caspofungin (or the EtOH solvent control) for 30 minutes, and GFP emission and CFW staining were assessed via flow cytometry. (A) Representative scatter plot and adjunct histograms for CFW staining and GFP emission of *mkc1Δ/Δ CHS3-GFP* cells treated with caspofungin. Gates represent populations of high and low chitin within the sample. (B) Representative scatter plot with adjunct histograms for CFW staining and GFP emission when plotting low (blue) and high (red) chitin populations independently. (C) GFP emission of cells within the low and high chitin populations following caspofungin exposure to *mkc1Δ/Δ CHS3-GFP* cells and normalization to median cell size for each of the two populations. (ns = not significant, by student's t-test)(n = 3 biological replicates).

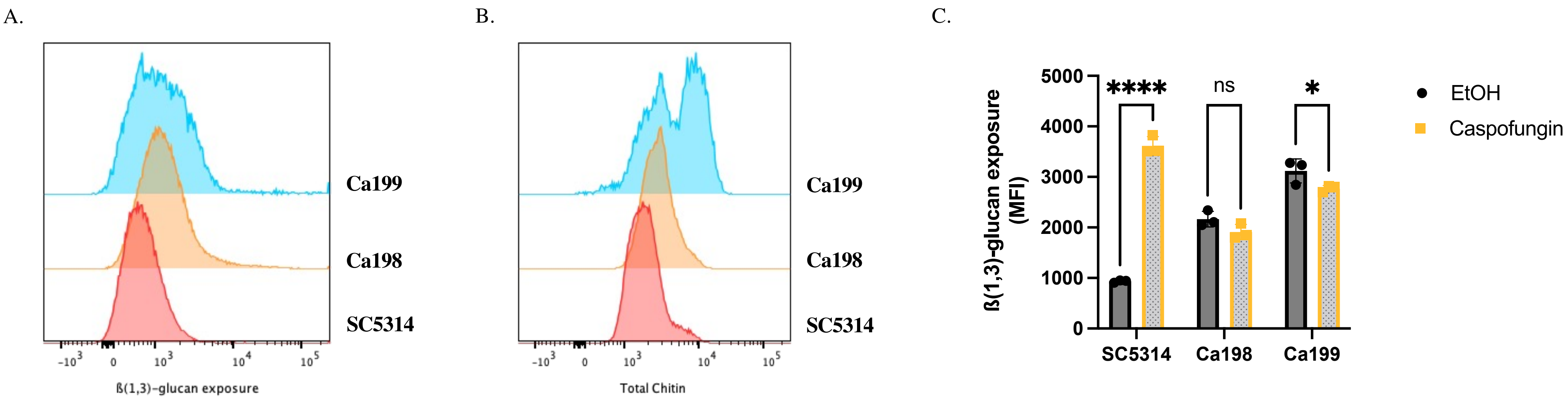
A.
B.
C.
Ca199
Ca198
SC5314
-10^3
0
10^3
10^4
10^5
ß(1,3)-glucan exposure
Total Chitin
ß(1,3)-glucan exposure (MFI)
5000
4000
3000
2000
1000
0
****
ns
*
EtOH
Caspofungin

Fig. S8: Caspofungin-induced unmasking in yeast cells requires drug-mediated inhibition of Fks1.

(A-C) The SC5314 derived *LEU2/leu2*Δ wild-type strain and two caspofungin resistant *C. albicans* isolates (Ca198 and Ca199) were grown to mid-log phase and exposed to 46.9 ng/ml of caspofungin (or EtOH solvent control) for 30 minutes. ß(1,3)-glucan exposure and total chitin levels were then assessed by flow cytometry. (A) Representative histogram for basal ß(1,3)-glucan exposure of mid-log phase cells treated with an ethanol solvent control. (B) Representative histogram for basal chitin levels of mid-log phase cells treated with an ethanol solvent control. (C) ß(1,3)-glucan unmasking of strains treated with an ethanol solvent control or caspofungin. (*$p<0.05$, ****$p<0.0001$, by students t-test).
