# Supplementary material for: Caspofungin-induced β(1,3)-glucan exposure in Candida albicans is driven by increased chitin levels
Source: mBio. 2023 Jun 28;14(4):e00074-23. doi: 10.1128/mbio.00074-23 (PMC10470516; doi:10.1128/mbio.00074-23)
Supplement: Table S1 — Strains used in this study. [file mbio.00074-23-s0002.docx]

**Table S1: Strains used in this study.**

| **Strain** | **Genotype** | **Parent** | **Source or Reference** |
| --- | --- | --- | --- |
| AWY006 | *LEU2/leu2∆* (Wild Type strain throughout this communication) | SC5314 | 1 |
| AWY203 | *mkc1Δ/Δ LEU2/leu2∆* | AWY006 | This Study |
| AWY245 | *CHS3/CHS3-GFP LEU2/leu2∆* | AWY006 | This Study |
| AWY307 | *mkc1Δ/Δ CHS3/CHS3-GFP LEU2/leu2∆* | AWY203 | This Study |
| Ca198/DPL21 | caspofungin resistant, fks1-S645P mutation | Clinical isolate | David Perlin |
| Ca199/ DPL1016 | caspofungin resistant, fks1-S645P mutation | Clinical isolate | David Perlin |

**References:**

1. Wagner, A. S. et al. Activation of Cph1 causes ß(1,3)-glucan unmasking in Candida albicans and attenuates virulence in mice in a neutrophil-dependent manner. PLoS Pathog 17, e1009839, doi:10.1371/journal.ppat.1009839 (2021).
