# Supplementary material for: Caspofungin-induced β(1,3)-glucan exposure in Candida albicans is driven by increased chitin levels
Source: mBio. 2023 Jun 28;14(4):e00074-23. doi: 10.1128/mbio.00074-23 (PMC10470516; doi:10.1128/mbio.00074-23)
Supplement: Table S2 — Primers used in this study. [file mbio.00074-23-s0003.docx]

**Table S2: Primers used in this study.**

| **Primer Name** | **Sequence (5' to 3')** | **Description** | **Source** |
| --- | --- | --- | --- |
| AHO1096 | GACGGCACGGCCACGCGTTTAAACCGCC | Fragment A forward primer for CRISPR-mediated gene deletion. | 1 |
| AHO1097 | CCCGCCAGGCGCTGGGGTTTAAACACCG | Fragment B reverse primer for CRISPR-mediated gene deletion. | 1 |
| AHO1098 | caaattaaaaatagtttacgcaag | Fragment A reverse primer for CRISPR-mediated gene deletion. | 1 |
| AHO1237 | aggtgatgctgaagctattgaag | Fragment C forward primer for CRISPR-mediated gene deletion. | 1 |
| AHO1238 | TGTATTTTGTTTTAAAATTTTAGTGACTGTTTC | Fragment C reverse primer for CRISPR-mediated gene deletion. | 1 |
| AWO242 | CGTAAACTATTTTTAATTTGAGAACCCCGACG  AAAATGCTGTTTTAGAGCTAGAAATAGC | *MKC1* gRNA forward primer for fragment B amplification for CRISPR-mediated gene cleavage | This Study |
| AWO244 | ATTTCCATAATGGATCAACAAGAAGCACCCA  TATATTATGGCAGATCCGTTAGTTGAAACAC  ACAACCACGTTATCCCGGGGTATGTTACTCTC  GAATTA | *MKC1* repair template for whole gene deletion (sense oligomer) | This Study |
| AWO245 | TAATTCGAGAGTAACATACCCCGGGATAACG  TGGTTGTGTGTTTCAACTAACGGATCTGCCAT  AATATATGGGTGCTTCTTGTTGATCCATTATG  GAAAT | *MKC1* repair template for whole gene deletion (antisense oligomer) | This Study |
| AWO246 | GATGTCACATCAACTTCAC | *MKC1* whole gene deletion check primer forward (Sits -560bp upstream from start codon in 5'UTR) | This Study |
| AWO247 | GAGACAAGAAGTATTATTTCATCTC | *MKC1* whole gene deletion check primer reverse (Sits +513bp from stop codon in 3'UTR) | This Study |
| AWO146 | CTTGGGATCCATCTAATACTGGGGGAAATT  TGATTGATGATTTAAGTCAAGGATCTTCTTC  AGGGTCCAGTggtggtggttctaaaggtgaagaattatt | GFP forward primer with 80bp overhang of *CHS3* (80bp upstream stop codon) | This Study |

**Table S2 Continued**

| **Primer Name** | **Sequence (5' to 3')** | **Description** | **Source** |
| --- | --- | --- | --- |
| AWO147 | TTTTTATATTGTTAATAATTTTATATAACCAT  ATACATAAATAAAAGTCCTTTCTCTCTCTCT  TTTTTAAGCTTTAACCCAAagaactagtggatccccc | GFP reverse primer with 80bp overhang of *CHS3* (80bp downstream stop codon) | This Study |
| AWO270 | CTATTTGTACAATTCATCCATAC | *CHS3-GFP* reverse primer for screening insertion at C-terminal end of *CHS3* (Sits at end of *GFP* ORF) | This Study |
| SLO12 | GCAGTTATTTTCGGGTTACCTGGTTGTT | *CHS3-GFP* forward check primer. (Sits in *CHS3* ORF -3247 bp downstream of start codon) | This Study |
| AWO401 | GATTGGTACTTTGGTGTTACC | *CHS3-GFP* forward primer. This is to amplify gDNA from WT *CHS3-GFP* for transformation into *mkc1Δ/Δ.* The primer sits in the *CHS3* ORF at 523bp upstream of the N-terminal start to the *GFP* ORF. | This Study |
| AWO402 | GTGTTTGTTATTGTCTCAAAACTG | *CHS3-GFP* reverese primer. This is to amplify gDNA from WT *CHS3-GFP* for transformation into *mkc1Δ/Δ.* The primer sits in the *CHS3* 3’ UTR at 443bp downstream of the C-terminal stop codon of the *GFP* ORF. | This Study |

**Refences:**

1. Nguyen N, Quail MMF, Hernday AD. An Efficient, Rapid, and Recyclable System for CRISPR-Mediated Genome Editing in Candida albicans. mSphere. 2017;2(2). Epub 2017/05/13. doi: 10.1128/mSphereDirect.00149-17. PubMed PMID: 28497115; PubMed Central PMCID: PMCPMC5422035.
