# Supplementary material for: Caspofungin-induced β(1,3)-glucan exposure in Candida albicans is driven by increased chitin levels
Source: mBio. 2023 Jun 28;14(4):e00074-23. doi: 10.1128/mbio.00074-23 (PMC10470516; doi:10.1128/mbio.00074-23)
Supplement: Table S3 — Plasmids used in this study. [file mbio.00074-23-s0004.docx]

**Table S3: Plasmids used in this study.**

| **Strain Name** | **Description** | **Marker** | **Parent**  **/Source** |
| --- | --- | --- | --- |
| pTB198 | *GFP-CaNAT* | Ampicillin, Nourseothricin | This Study |
| pADH110 | CRISPR fragment A template | Ampicillin, Nourseothricin* | (76) |
| pADH119 | CRISPR fragment B template | Ampicillin, Nourseothricin* | (76) |
| pADH137 | CRISPR Cas9 expression Plasmid | Ampicillin, Nourseothricin* | (76) |

*These plasmids each function together to make a split nourseothricin gene that comes together to form a functional open reading frame after successful transformation and recombination into the *Candida albicans LEU2* locus.

**References:**

1. Nguyen N, Quail MMF, Hernday AD. An Efficient, Rapid, and Recyclable System for CRISPR-Mediated Genome Editing in Candida albicans. mSphere. 2017;2(2). Epub 2017/05/13. doi: 10.1128/mSphereDirect.00149-17. PubMed PMID: 28497115; PubMed Central PMCID: PMCPMC5422035.
