# Supplementary material for: Caspofungin-induced β(1,3)-glucan exposure in Candida albicans is driven by increased chitin levels
Source: mBio. 2023 Jun 28;14(4):e00074-23. doi: 10.1128/mbio.00074-23 (PMC10470516; doi:10.1128/mbio.00074-23)
Supplement: Text S1 — Growth conditions and cloning methods used in this study. [file mbio.00074-23-s0005.docx]

**Text S1: Growth conditions and cloning methods used in this study.**

**Growth media and culture conditions.** *C. albicans* strains were grown in YPD media (1% yeast extract, 2% peptone, 2% dextrose) while shaking at 225rpm at 30°C (1). Minimal media (0.67% yeast nitrogen base without amino acids, 2% dextrose, 2% agar) (1) was used to select for removal of the integrated CRISPR/Cas9 cassette following gene deletion (2).

**Plasmid and strain construction**

All *C. albicans* mutant strains (Table S1) were generated with the use of CRISPR-Cas9 as previously described (2). All plasmids and primers used to create each mutant can be found in Table S2 and Table S3, respectively.

The *CHS3-GFP* mutant in the SC5314 derived *LEU2/leu2∆* wild-type background was generated by amplifying an ~5,500 bp fragment of *GFP-NAT* from pTR198 using primers AWO146 and AWO147, which introduce 80 bp of homology to the regions immediately upstream and downstream of the *CHS3* stop codon in the genome to each side of the PCR fragment. The PCR product was gel purified with the use of the QIAquick Gel Extraction Kit (Qiagen) and the purified fragment was then transformed via electroporation into the SC5314 *C. albicans* strain as previously described (3). Successful transformants were selected on YPD plates containing 200μg/ml nourseothricin.

The *mkc1Δ/ΔCHS3-GFP* double mutant was generated by amplifying the GFP fragment from purified SC5314 *CHS3-GFP* genomic DNA using primers AWO401 and AWO402. This design amplified 523 bp of 5’ homology to the *CHS3* open reading frame (ORF) immediately upstream of the start of the GFP open reading frame and 443 bp of the *CHS3* 3’ untranslated region (UTR) immediately following the GFP stop codon and NAT1 marker to generate a PCR product that was ~5,000 bp in size. Following PCR amplification, the 5,000 bp PCR product was gel purified and then transformed via electroporation into the *mkc1Δ/Δ* mutant as previously described (3). Successful transformants were selected on YPD plates containing 200 μg/ml nourseothricin.

**References**

1. Styles C. 2002. How to set up a yeast laboratory. Methods Enzymol 350:42-71.

2. Nguyen N, Quail MMF, Hernday AD. 2017. An Efficient, Rapid, and Recyclable System for CRISPR-Mediated Genome Editing in Candida albicans. mSphere 2.

3. Chen YL, Montedonico AE, Kauffman S, Dunlap JR, Menn FM, Reynolds TB. 2010. Phosphatidylserine synthase and phosphatidylserine decarboxylase are essential for cell wall integrity and virulence in Candida albicans. Mol Microbiol 75:1112-32.
